# Integrated Multi-Omics Reveals the Developmental Programs and Metabolic Regulation of the Rare Edible Fungus *Buchwaldoboletus xylophilus*

**DOI:** 10.3390/biology15161343

**Published:** 2026-08-08

**Authors:** Yuying Liu, Zhiyuan Jia, Teng Jiang, Na Zhang, Lixiao Song, Jin Zhang, Xiaolei Wan, Haiqiang Wang, Can Du, Daoyin Shen, Minglei Li, Jianzhao Qi

**Affiliations:** 1 Shaanxi Key Laboratory of Natural Products & Chemical Biology, College of Chemistry & Pharmacy, Northwest A&F University, Yangling, Xianyang 712100, China; 2 Center of Edible Fungi, Northwest A&F University, Yangling, Xianyang 712100, China; 3 Yangling Zhijun Fungi Biotechnology Engineering Co., Ltd., Yangling 712100, China; 4 Department of Bioengineering, Shaanxi Agriculture and Forestry Technology University, Yangling, Xianyang 712100, China

**Keywords:** *Buchwaldoboletus xylophilus*, transcriptome, non-targeted metabolomics, O2PLS, WGCNA, alternative splicing, ceRNA network, KEGG pathway enrichment

## Abstract

*Buchwaldoboletus xylophilus* is a rare saprotrophic edible mushroom in the Boletaceae and is the second bolete species to have been successfully cultivated artificially, yet its developmental molecular regulation remains unexplored. We integrated RNA-seq transcriptomics and untargeted LC-MS/MS metabolomics across five developmental stages (A–E), applying differential expression, alternative splicing, ceRNA network construction, WGCNA, machine learning, consensus clustering, and O2PLS joint modelling. Transcriptomic analysis revealed that the mycelium-to-primordium transition (stage A to C) showed the most drastic reprogramming, with 2233 differentially expressed genes, while metabolomic comparison between stage A and E revealed overwhelmingly upregulated metabolites (184 up vs. 10 down), indicating asymmetric regulation between the two omics layers. Notably, mutually exclusive exon splicing events exhibited high functional significance, and all top ceRNA hubs were lncRNAs. Unsupervised consensus clustering validated the molecular distinctness of the sampled stages. Metabolite enrichment highlighted antioxidant functions and ABC transporter pathways, and O2PLS integrative modelling confirmed a strong shared developmental signal between transcriptome and metabolome. This study provides the first multidimensional molecular atlas for *B. xylophilus*, revealing a non-linear developmental programme, transcriptome–metabolome decoupling, and a central role of antioxidant defence throughout development. Our findings offer mechanistic clues regarding the evolutionary transition from ectomycorrhizal to saprotrophic lifestyles in the Boletaceae and establish a multi-omics foundation for the molecular breeding of this emerging edible and medicinal mushroom.

## Simple Summary

Many edible mushrooms are difficult to cultivate artificially because they depend on living hosts. The rare edible bolete *Buchwaldoboletus xylophilus* (Petch) Both & B. Ortiz is a notable exception, as its saprotrophic lifestyle makes it amenable to artificial domestication. However, the molecular mechanisms guiding its orderly development from a simple mycelial network into a fully formed fruiting body have long remained elusive. In this study, we integrated transcriptomic and metabolomic data from five developmental stages to construct the first systematic molecular atlas of this fungus’s life cycle. Our results reveal that the transition from early mycelium to primordium (the young fruiting body) represents the most drastic phase of molecular reprogramming, involving the differential expression of more than one-third of all genes. Strikingly, while the overall numbers of up- and down-regulated genes remained roughly balanced throughout development, metabolite levels exhibited a marked asymmetry—184 compounds increased in abundance, whereas only 10 decreased. This imbalance suggests that, beyond transcriptional control, the fungus relies heavily on post-transcriptional or translational regulatory layers. Furthermore, natural antioxidant compounds played a central protective role at every developmental stage, underscoring the critical importance of maintaining redox homeostasis. These findings not only elucidate the molecular logic underlying fruiting-body construction in this saprotrophic mushroom but also provide a genetic blueprint that can guide breeding efforts to enhance yield, improve quality, and optimize cultivation practices, thereby facilitating the broader adoption of this emerging cultivable edible mushroom.

## 1. Introduction

Edible fungi have become an increasingly prominent focus in food science and natural product research, owing to their rich nutritional value and distinctive bioactive constituents. Fruiting body (or sclerotia) development in macrofungi (mushrooms) represents one of the most striking morphogenetic processes in nature, entailing the transition from a simple filamentous mycelium to a complex three-dimensional multicellular structure [1,2]. This process is under precise multi-level regulation encompassing gene transcription, post-transcriptional regulation, post-translational modification, and metabolic networks. The great majority of known edible mushrooms belong to the class Agaricomycetes within the phylum Basidiomycota. Studies on model species such as *Coprinopsis cinerea* and *Schizophyllum* commune have identified a large repertoire of genes and transcription factors associated with fruiting body formation, including homeodomain transcription factors, C2H2 zinc finger proteins, GATA factors, and the Velvet complex, among others [3]. However, research into the developmental regulatory mechanisms of non-model fungi, particularly edible mushrooms of significant economic importance, remains extremely limited [3].

The Boletaceae is one of the most species-rich and ecologically diverse families within the Basidiomycota, comprising over 1000 species [4]. The vast majority of species in this family form ectomycorrhizal (ECM) symbioses with trees and play a pivotal role in carbon and nitrogen cycling within forest ecosystems. Comparative genomic studies have revealed that the genomes of ECM boletes (30–71 Mbp) have undergone substantial transposable element (TE) expansion and loss of genes encoding lignocellulose-degrading enzymes (CAZymes), reflecting an evolutionary transition from saprotrophy to symbiosis [5]. Nevertheless, a small number of bolete species have independently evolved a non-mycorrhizal (saprotrophic) lifestyle. This evolutionary reversal has conferred upon them the ability to grow independently of a host tree, which is the fundamental reason underpinning their artificial cultivability.

*Buchwaldoboletus xylophilus* (Petch) Both & B. Ortiz, a saprotrophic macrobasidiomycete belonging to the subfamily Chalciporoideae within the Boletaceae, is also commonly known as ‘Huangjianshouqing’ (Yellow Bluing Bolete) in China owing to its characteristic bruising reaction. The species was originally described as Boletus xylophilus by Petch in 1922 from Sri Lanka, and was subsequently transferred to the genus *Buchwaldoboletus* following the systematic revision by Ortiz-Santana and Both (2011) [1]. Molecular phylogenetic analyses based on multiple gene markers, including ITS, LSU, and RPB2, place *B. xylophilus* firmly within the subfamily Chalciporoideae—one of the earliest-diverging subfamily-level clades of the Boletaceae. *Buchwaldoboletus xylophilus* is distributed across tropical regions of Asia (Sri Lanka, Malaysia, the Philippines, Yunnan and Hainan in China, and Kerala in India), with ITS sequence similarity as high as 99.71% between Chinese and Indian specimens. In 2021, a joint team from the Yunnan Institute of Tropical Crops and Hainan Medical University successfully achieved artificial cultivation of *B. xylophilus* in mushroom growing rooms, making it the second bolete species worldwide—after *Phlebopus portentosus*—to be brought into cultivation [4,5,6].

*Buchwaldoboletus xylophilus* occupies a pivotal phylogenetic position, displays unique saprotrophic ecological adaptations, and harbours considerable potential for commercial exploitation. A reference genome for this species has recently been released, providing foundational genomic resources for dissecting its biological and molecular mechanisms. However, a static genome sequence is inherently limited in its ability to capture the dynamic shifts in gene expression and the rewiring of metabolic networks that unfold during development. Genomic information alone is therefore insufficient to systematically decode the molecular regulatory logic that governs progression from mycelial growth to fruiting body formation. To bridge this gap, the present study sampled five representative developmental stages and performed RNA-seq-based transcriptomic profiling, non-targeted LC-MS/MS metabolomic profiling, and an integrative multi-omics analysis. Transcriptomic analyses centred on 6435 protein-coding genes and encompassed differential expression analysis, alternative splicing detection, ceRNA network inference, and WGCNA. Metabolomic profiling detected 3204 features, of which 1835 were annotated via spectral matching and accurate mass-based identification. By applying four complementary integration strategies—O2PLS, Pearson pairwise correlation, nine-quadrant diagram analysis, and module–metabolite association—we revealed that the developmental programme follows a nonlinear trajectory: transcriptomic and metabolic reprogramming was most pronounced from stages A to C, whereas post-transcriptional regulation predominated in the D–E transition. Moreover, a striking asymmetry between transcriptomic and metabolomic changes was observed: in the A vs. E comparison, 184 metabolites were up-regulated versus only 10 down-regulated, while the numbers of up- and down-regulated genes were approximately equal. Antioxidant-related metabolites carried substantial discriminative weight in stage-classification models. Collectively, this study uncovers the cross-level regulatory principles underpinning development in *B. xylophilus* and delivers a multi-omics framework that can inform future molecular breeding efforts and the optimisation of artificial cultivation.

## 2. Materials and Methods

### 2.1. Experimental Materials and Sample Collection

*Buchwaldoboletus xylophilus* was cultivated in the edible mushroom artificial cultivation chambers of Yangling Zhijun Fungi Biotechnology Engineering Co., Ltd., (Xianyang, China). The cultivation substrate was formulated with 20% broadleaf wood sawdust, 30% cottonseed hulls, 20% wheat grains, 10% maize flour, 10% red soil, 8% wheat bran, 1% gypsum powder, 1% glucose, 0.1% KH_2_SO_4_, and 0.1% FeSO_4_ and sterilised by steaming at atmospheric pressure. For liquid medium preparation, 100 g of the sterilised solid substrate was combined with 1 L of deionised water, boiled for 30 min, and filtered. The filtrate was mixed with potato dextrose broth (PDB) at a 1:1 (*v*/*v*) ratio and sterilised by autoclaving at 121 °C for 30 min, and this liquid medium was used for mycelial activation. Solid substrate of the same formulation was then inoculated with the activated liquid culture to induce primordium formation and fruiting body maturation.

**Figure 1:**
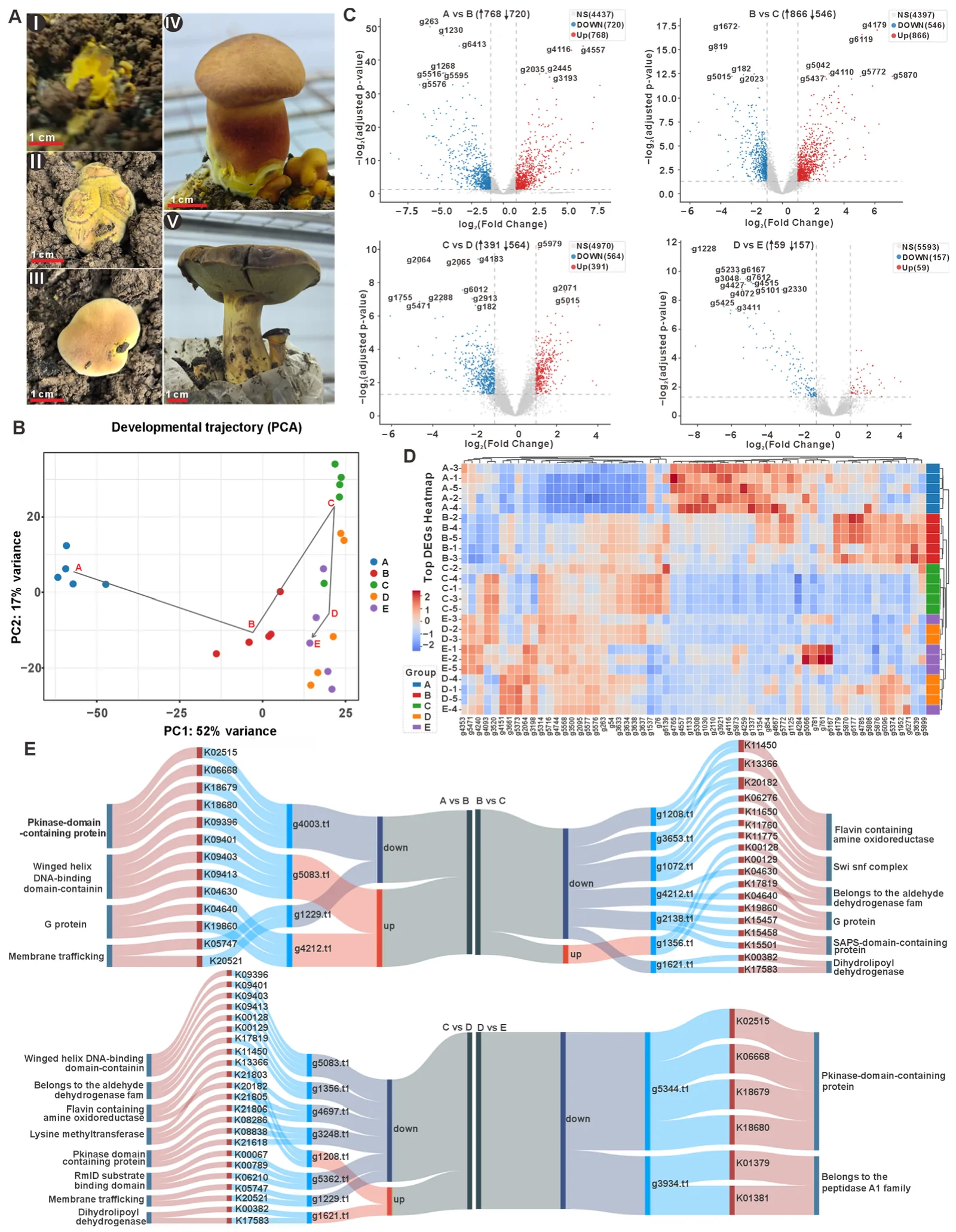
Morphological characteristics and global transcriptomic profiles across five developmental stages of *B. xylophilus*. (**A**) The five designated developmental stages (I–V) correspond one-to-one with stages A–E. (**B**) Principal component analysis (PCA) score plot based on the TMM-normalized expression matrix of 6435 protein-coding genes. The proportions of variance explained by PC1 and PC2 are indicated on each axis. Ellipses represent 95% confidence intervals. Samples are ordered along the PC1 axis according to developmental stages A to E. (**C**) Volcano plots of differentially expressed genes (DEGs) from four adjacent stage comparisons (A vs. B, B vs. C, C vs. D, D vs. E). The x-axis shows log_2_ fold change, and the y-axis shows −log_10_(FDR-adjusted *p*-value). Red dots indicate significantly upregulated genes (log_2_FC > 1, FDR < 0.05), blue dots indicate significantly downregulated genes (log_2_FC < −1, FDR < 0.05), and gray dots represent non-significant genes. The total number of significant DEGs (up/down) is annotated for each comparison. (**D**) Hierarchical clustering heatmap of the top 100 DEGs ranked by |log_2_FC| across the 10 stage comparisons. Rows represent genes, and columns represent comparison pairs (labeled at the bottom). The color scale indicates Z-score-standardized log_2_ fold change: red = upregulation; blue = downregulation. (**E**) Sankey diagram of KEGG enrichment results for DEGs in each developmental stage comparison.

Based on morphological characteristics, representative samples were collected at five developmental stages (Figure 1A): Stage A (vegetative mycelial stage), harvested at 3 days post liquid inoculation; Stage B (primordium formation stage), harvested at 6 days post substrate inoculation; Stage C (primordium growth stage), harvested at 9 days post substrate inoculation, when tiny white primordia were visible on the substrate surface; Stage D (fruiting body differentiation stage), harvested at 11 days post substrate inoculation, by which time cap and stipe structures had clearly differentiated; and Stage E (fruiting body maturation stage), harvested at 14 days post substrate inoculation, when caps had fully expanded and spore discharge had commenced. For each stage, five biological replicates were collected, with each replicate derived from an independent cultivation container. All samples were snap-frozen in liquid nitrogen immediately after collection and stored at −80 °C until further processing. Aliquots from the same sample batch were used separately for RNA extraction and metabolite extraction.

### 2.2. RNA-Seq Library Construction and Transcriptome Sequencing and Analysis

Total RNA was extracted using TRIzol reagent (Invitrogen, Carlsbad, CA, USA) according to the manufacturer’s instructions. RNA purity and concentration were measured with a NanoDrop 2000 spectrophotometer (Thermo Fisher Scientific, Waltham, MA, USA), and RNA integrity number (RIN) was assessed using an Agilent 2100 Bioanalyzer (Agilent Technologies, Santa Clara, CA, USA); all samples had RIN > 7.0. For each sample, 1 μg of total RNA was used for library construction. Strand-specific libraries were prepared with the NEBNext Ultra II Directional RNA Library Prep Kit for Illumina (NEB, Ipswich, MA, USA) following the poly(A) enrichment protocol. Library quality was verified on an Agilent 2100 Bioanalyzer, and effective library concentration was accurately quantified via qPCR using the KAPA Library Quantification Kit (Roche, Basel, Switzerland). Libraries were sequenced on the Illumina NovaSeq 6000 platform (Illumina, San Diego, CA, USA) in paired-end 150 bp mode, targeting approximately 6 Gb of clean reads per sample.

The reference genome of *B. xylophilus* (NCBI Taxonomy ID: 2820146, Genome accession No. GCA_054960005.1) was used. Ab initio gene prediction was performed with AUGUSTUS (v3.4.0) using gene models from the closely related species *Phlebopus portentosus* and *Boletus edulis* as training sets, yielding 6435 protein-coding genes [7]. Raw sequencing data were subjected to quality assessment with FastQC (v0.11.9) [8], followed by the removal of Illumina adaptor sequences and low-quality bases (Phred score < 20, sliding window 4 bp) using Trim Galore (v0.6.7, wrapping Cutadapt and FastQC). The resulting clean reads were aligned to the reference genome with HISAT2 (v2.2.1, -rna-strandness RF) [9]. Gene expression levels were quantified using featureCounts (v2.0.1, -s 2-t exon-g gene_id) based on the AUGUSTUS gene annotation [10], generating a raw count matrix for 25 samples (five developmental stages × five biological replicates).

Downstream bioinformatics analyses were conducted using an in-house Comprehensive Fungal Transcriptome Pipeline v5 (built upon R v4.2 [11] and Bioconductor v3.16); all scripts have been deposited on GitHub on 30 June 2026 (https://github.com/Waramaforlong/IMPFD). The expression matrix was subjected to TMM normalisation and differential expression analysis with DESeq2 (v1.36.0) [12]. Pairwise comparisons were performed for ten developmental stage combinations, and differentially expressed genes (DEGs) were identified using thresholds of |log_2_ fold change| > 1 and Benjamini–Hochberg-adjusted FDR < 0.05. Five types of differential alternative splicing events—SE, MXE, alternative 5′ splice site (A5SS), alternative 3′ splice site (A3SS), and retained intron (RI)—were detected using rMATS (v4.1.0, -t paired), with significance cut-offs of FDR < 0.05 and |ΔPSI| > 0.1 [13]. RNA editing sites were identified with REDItools (v2.0) following GATK best practices, focusing on A-to-G (A-to-I) editing events [14]. Differential splicing frequency analysis at individual exon–exon junctions was carried out within a generalised linear model framework.

Competing endogenous RNA regulatory networks were constructed as follows: miRNA–mRNA and miRNA–lncRNA target relationships were predicted with psRNATarget (2017 release) (expectation value ≤ 3.0) [15]; RNA pairs sharing at least five common miRNAs were selected as candidate ceRNA pairs; Pearson expression correlation coefficients were calculated for each candidate pair; pairs showing significant positive correlations (r > 0.8, *p* < 0.05) were retained as final ceRNA interactions. Network topology and visualisation were accomplished with the igraph R package [16]. WGCNA (v1.72) was used to construct gene co-expression networks [17]: the top 25% most variable genes (*n* = 1608) were selected, a soft-thresholding power of 20 (chosen based on scale-free topology fit R^2^ > 0.85) was applied to build a weighted adjacency matrix, and co-expression modules were detected using topological overlap matrix (TOM)-based hierarchical clustering with dynamic tree cutting (minModuleSize = 30, mergeCutHeight = 0.25).

Machine learning classification was carried out using eight supervised learning algorithms within the caret R package (v6.0) [18]: random forest (RF), support vector machine with radial basis function kernel (SVM-RBF), extreme gradient boosting (XGBoost) [19], extremely randomised trees (Extra Trees), adaptive boosting (AdaBoost), K-nearest neighbours (KNNs), gradient boosting machine (GBM), and multinomial logistic regression (MLP). All models were evaluated via five-fold cross-validation using accuracy and Cohen’s Kappa as performance metrics. Recursive feature elimination (RFE, with RF as the base learner) was employed to select core discriminative gene sets. Consensus clustering-based molecular subtyping was performed with ConsensusClusterPlus (v1.58.0, 1000 resampling iterations, 80% sample fraction, k = 2–6, hierarchical clustering, distance metric = 1 − Pearson correlation coefficient) [20].

Functional annotation of genes was performed with EggNOG-mapper (v2.1.9) against the eggNOG 5.0 database [21], resulting in annotation for 4751 genes (73.8%). KEGG orthologue (KO) enrichment analysis was carried out using a hypergeometric test, with all EggNOG-annotated genes serving as the background set; *p*-values were corrected by the Benjamini–Hochberg procedure. Functional annotation of transcription factors and developmental regulatory genes was guided by the compendia of fungal developmental regulators compiled by Nowrousian [22] and Sun et al. [23]. Visualisation was predominantly performed using ggplot2 (v3.4.0) [24] and ComplexHeatmap (v2.12.0) [19].

### 2.3. Non-Targeted Metabolomic Profiling and Analysis Based on LC-MS/MS

Metabolites were extracted with ethyl acetate from the fermentation broth of *B. xylophilus* at the mycelial stage and from fruiting bodies at different developmental stages separately. The extracts were concentrated under reduced pressure to obtain crude extracts, which were then reconstituted in LC-MS grade methanol, adjusted to a concentration of 30 μg/mL, and filtered through a 0.22 μm organic nylon membrane before being transferred to sample vials. Metabolomic data were acquired on an AB X500R QTOF liquid chromatography–mass spectrometry system (SCIEX, Marlborough, MA, USA). Chromatographic separation was carried out on an Agilent Infinity Lab Poroshell 120 EC-C column (2.1 × 150 mm, 2.7 μm, Agilent Technologies, Inc., Santa Clara, CA, USA) maintained at 40 °C. The mobile phases consisted of water (A) and methanol (B). The gradient elution programme was as follows: 0–10 min, 10% B; 10–25 min, 10% → 100% B; 25–27 min, 100% B; 27–28.5 min, 100% → 10% B; 28.5–30 min, 10% B (column re-equilibration). The flow rate was 0.3 mL/min and the injection volume was 5.0 μL. The mass spectrometer was equipped with an electrospray ionisation (ESI) source and operated in positive/negative ion switching mode using information-dependent acquisition (IDA). Positive ion mode parameters: m/z scan range 60–1200, collision energy +10 eV, source temperature 400 °C, curtain gas 35 psi, nebuliser gas 55 psi, and ion spray voltage +5500 V. Negative ion mode parameters: m/z scan range 60–1500, collision energy −10 eV, source temperature 400 °C, curtain gas 35 psi, nebuliser gas 55 psi, and ion spray voltage −4500 V. Raw data were acquired with Analyst TF 1.7.1 software and converted to the open-format mzML (centroid mode) using ProteoWizard msconvert (v3.0.22102) [25].

Non-targeted metabolomic feature detection was performed using an in-house two-dimensional binning algorithm based on pymzml (v2.5.6). The processing steps were as follows: (1) all centroid peaks (*m*/*z*, RT, intensity) were extracted from each mzML file; (2) a two-dimensional bin grid was constructed with an m/z tolerance of 10 ppm and an RT tolerance of 0.3 min; (3) isotopic and adduct peaks within each bin were merged, and the peak with the highest intensity was retained as the representative peak; (4) a noise threshold of absolute intensity > 5000 counts was applied, and a feature was required to be present in at least two samples; (5) only features with a coefficient of variation (CV) < 30% in QC samples were retained as reliably quantifiable features. A total of 3204 metabolic features were ultimately detected (2729 in ESI+ mode, 85.1%; 475 in ESI− mode, 14.9%).

### 2.4. Metabolite Identification

Metabolite identification was performed using a dual-strategy complementary approach aligned with the Metabolomics Standards Initiative (MSI) confidence grading system [26]. Strategy 1 employed MS^2^ cosine spectral matching (MSI Level 2 identification), wherein all MS^2^ spectra were extracted from the data-dependent acquisition (DDA) cycles of mzML files and subjected to preprocessing, including noise removal (discarding fragment ions with relative intensity < 1%), deconvolution, and intensity normalization, after which the processed spectra were matched against public spectral libraries GNPS [27] and MoNA [28] using cosine similarity with criteria comprising cosine similarity ≥ 0.2, precursor ion tolerance ±20 ppm, fragment ion tolerance ±20 ppm, and a minimum of three matched fragment ions, successfully annotating 92 metabolites all detected in ESI^−^ mode and representing 19.4% of features in that ionization mode. Strategy 2 utilized accurate mass matching (MSI Level 3 identification), where the remaining features were annotated by searching precursor ion accurate m/z values (±10 ppm) against a composite database integrating GNPS, MoNA, HMDB (v5.0) [29], and LipidBlast [30]. The combined strategies annotated a total of 1835 features (overall annotation rate 57.3%), with 1620 features (50.6% of all detected features) assigned InChIKey identifiers, while metabolite functional classification employed a 13-category fungus-specific keyword system encompassing polyketides, terpenoids, non-ribosomal peptides, alkaloids, amino acids, lipids, carbohydrates, nucleotides, TCA cycle/energy metabolism, phenylpropanoids, vitamins/cofactors, organic acids, and indole derivatives. Metabolomic data preprocessing and statistical analysis: log_2_-PQN (probabilistic quotient normalisation) was applied to eliminate systematic inter-sample variation [31], and robust spline correction (RSC) of QC samples was performed to minimise intra-batch signal drift. OPLS-DA modelling was conducted using the ropls R package (one predictive component plus one orthogonal component, validated with 200 permutation tests) [32]. Differentially accumulated metabolites were identified by Welch’s *t*-test with Benjamini–Hochberg (BH) FDR multiple-testing correction, using thresholds of |log_2_FC| > 1 and FDR < 0.05. Random forest classification was performed with the randomForest R package (ntree = 500, 70%/30% random split into training and test sets), and SVM-RBF served as an independent validation model. KEGG pathway mapping followed the workflow InChIKey → PubChem REST API → PubChem CID → KEGG REST API → KEGG Compound ID → KEGG Pathway ID [33]. Pathway enrichment was evaluated using a count-based ranking approach, i.e., the number of metabolites assigned to each pathway was counted and ranked, rather than hypergeometric test-based *p*-values, because a well-defined background set of detectable compounds is lacking in non-targeted metabolomics.

### 2.5. Transcriptome–Metabolome Integration Analysis

Integrative analysis was performed on 25 common samples (five developmental stages and five biological replicates, with transcriptomic and metabolomic samples paired one-to-one). Prior to integration, the input matrices were preprocessed: the top 2000 highly variable genes by expression variance (31.1% of all protein-coding genes) were selected from the transcriptome, and the top 500 highly variable metabolites by variance (27.2% of annotated features) were selected from the metabolome; both datasets were then mean-centred. To systematically elucidate the coordinated regulation between the transcriptome and the metabolome, four complementary multivariate integration strategies were employed: (1) O2PLS joint latent variable modelling was carried out using the OmicsPLS R package (v2.0.1) [34] with parameters *n* = 2 (two joint components), nx = 1 (one orthogonal component for the transcriptome), and ny = 1 (one orthogonal component for the metabolome) [35]. Joint score matrices (T and U), orthogonal scores, and loading matrices (W and C) were extracted, and variance decomposition was performed to quantify the proportions of joint, orthogonal, and noise contributions to the total variation in each omics layer. (2) Pearson correlation network analysis was conducted by computing Pearson correlation coefficients for all 1,000,000 gene–metabolite pairs (2000 × 500). After BH-FDR correction, pairs with |r| > 0.8 and FDR < 0.001 were considered strongly associated, and the global co-variation pattern was visualised using a two-way hierarchical clustering heatmap. (3) Nine-quadrant diagram analysis was performed by plotting gene log_2_FC against metabolite log_2_FC for the A-to-E stage comparison, and partitioning gene–metabolite pairs into nine quadrants according to the direction of change, thereby quantifying the degree and direction to which transcriptional changes drive metabolic changes. (4) WGCNA module–metabolite association analysis was performed by using the eigengene (first principal component) of each of the 12 gene co-expression modules identified by WGCNA as a functional unit, computing Pearson correlation coefficients between module eigengenes and individual metabolite abundances, and constructing a module–metabolite association heatmap. The aforementioned analytical methods have been integrated into a comprehensive analysis platform; for more details, please refer to WWWWW.

## 3. Results

### 3.1. Global Transcriptomic Features and Developmental Trajectory

Based on the expression matrix of 6435 protein-coding genes, TMM normalisation was applied to the 25 samples across five developmental stages, followed by principal component analysis (PCA) (Figure S1). The results showed that the samples were strictly ordered along the first principal component (PC1) axis according to developmental stage (A to E), rather than being randomly scattered. On the PC1 axis, the early mycelial stages (A and B) and the late fruiting body stages (D and E) were positioned at opposite ends and were completely separated. Stage C (primordium formation) was located in a transitional region of the PCA space, adjacent to the A–B group and progressively approaching the D–E group, accurately reflecting the biological character of the primordium as a developmental transition from mycelium to fruiting body (Figure 1B). The five biological replicates of each stage clustered tightly in PCA space. Stages A and E exhibited the highest within-group reproducibility, while stages B, C, and D showed slightly larger within-group variation, which was nevertheless significantly smaller than the between-stage differences (PERMANOVA, *p* < 0.001) (Figure S1). The PC1–PC2 plane explained the major proportion of the total transcriptional variation, indicating that the dominant biological factor driving global transcriptomic variation was the developmental programme, rather than biological replicates, batch effects, or technical noise (Figure 1C).

Systematic pairwise differential expression analysis was performed across the five developmental stages (DESeq2, |log_2_FC| > 1, FDR < 0.05) (Figure 1C). The numbers of differentially expressed genes (DEGs) displayed a markedly nonlinear distribution (Table 1). The comparison between Stage A and Stage C identified 2233 DEGs, representing 34.7% of all protein-coding genes—the largest number among all ten pairwise comparisons and significantly exceeding any other comparison between adjacent stages. In this comparison, up-regulated genes (1281, 57.4%) substantially outnumbered down-regulated genes (951, 42.6%), indicating that the developmental transition from mycelium to primordium involves a large-scale transcriptional activation event that mobilises over one-third of the genome. In contrast, the overall comparison between Stage A and Stage E (the start- and endpoints of development) identified 1917 DEGs (29.8%), with nearly equal numbers of up- and down-regulated genes (988 up, 928 down) (Figure 1C). This markedly contrasts with the pronounced up-regulation asymmetry observed in the Stage A to C transition, suggesting complex oscillatory gene expression dynamics throughout development, in which a subset of genes activated during the Stage A to C transition may be re-suppressed at later developmental stages.

Among adjacent-stage comparisons, the transcriptional changes from A to B (1489 DEGs, 23.1%) and B to C (1413 DEGs, 22.0%) were of comparable magnitude; however, they differed fundamentally in EggNOG functional enrichment categories (see Section 3.3 for details), reflecting a functional shift from growth to differentiation. The D-to-E comparison yielded the fewest DEGs (217, only 3.4% of the genome) and was dominated by down-regulated genes (59 up, 158 down; an up-to-down ratio of approximately 0.37), indicating that the transition from fruiting body maturation to sporulation is accomplished through limited transcriptional fine-tuning rather than large-scale reprogramming.

Non-adjacent comparisons spanning multiple developmental stages further highlighted the nonlinear trajectory of development: the cumulative transcriptional divergence from stage C to E (primordium to spore maturation, 1810 DEGs, 28.1%) was nearly comparable to that observed across the entire developmental program from A to E (1917 DEGs), whereas the B-to-D comparison (mycelial expansion to fruiting body differentiation, encompassing two developmental intervals) yielded substantially fewer DEGs (690, 10.7%) than the single-step A-to-C transition (2233 DEGs). Collectively, these findings converge on a central conclusion: the developmental transcriptional program of *B. xylophilus* is not governed by a monotonic relationship wherein greater developmental distance invariably corresponds to greater transcriptional divergence but rather is organized into a highly nonlinear, non-monotonic architecture centered around pivotal developmental milestones—most prominently primordium formation.

Hierarchical clustering heatmap of the top 100 differentially expressed genes (DEGs) ranked by |log_2_FC| across the five developmental stages revealed a sample dendrogram highly congruent with the developmental stages, with no cross-stage sample misassignment observed (Figure 1D). This result indicates that developmental stage is the dominant driver of expression variation and that batch effects are negligible.

Further EggNOG functional enrichment analysis was performed on the 4751 annotated genes (hypergeometric test, BH-FDR correction), revealing the functional systems differentially activated across developmental transitions [21]. In the comparison between the Stage A and Stage C, which yielded the largest number of DEGs, the most significantly enriched functional categories included the flavin amine oxidoreductase family (K20182, K13366, and K11450, potentially involving in redox signalling during primordium formation), terpene synthases (K12249 and K22060, indicating activation of secondary metabolite biosynthesis), the ABC transporter PDR subfamily (K08712, with pleiotropic drug resistance transporters mediating the transmembrane transport of secondary metabolites), and cytochrome P450 monooxygenases (K10437, catalysing the oxidative modification of secondary metabolites). The co-enrichment of terpene synthases, P450s, and ABC transporters constituted a functional triad—secondary metabolite biosynthesis, oxidative modification, and transmembrane transport—strongly suggesting that primordium formation is not merely a morphological turning point, but also a molecular switch that triggers large-scale activation of secondary metabolic pathways, consistent with the known roles of quorum-sensing signals and developmentally regulated secondary metabolites in fungi [22,23].

In the overall comparison from the start point (Stage A) to the end point (Stage E) of development, the enrichment pattern revealed a different theme, prominently featuring the peptidase A1 family (K01379, K01381; involved in protein degradation and amino acid recycling), glycoside hydrolase families (K19356/GH61, K18576/GH12, K21850/GH16; catalysing cellulose and hemicellulose hydrolysis, reflecting saprotrophic carbon acquisition), the aldehyde dehydrogenase family (K00128; involved in aldehyde oxidative detoxification), and the multicopper oxidase family (K19791; potentially involved in lignin degradation and iron metabolism) (Figure S2A). Collectively, these enzyme families point to a sustained requirement for active carbon acquisition, protein turnover, and redox homeostasis throughout fruiting body development. Enrichment in the comparison between Stage A and Stage B (1489 DEGs) was biased towards ribosome biogenesis and basal metabolism, characteristic of vegetative growth (Figure S2B), whereas enrichment in the comparison between Stage B and Stage C (1413 DEGs) was skewed towards signal transduction and secondary metabolism (Figure S2C). This complementary pattern reveals a dual role for Stage B as a critical developmental node: it represents both a continuation of vegetative growth and a ‘pre-differentiation’ phase that primes the signalling and metabolic foundations for primordium formation.

### 3.2. Machine Learning Classification, Core Regulatory Factor Prediction, and Co-Expression Network Construction

**Figure 2:**
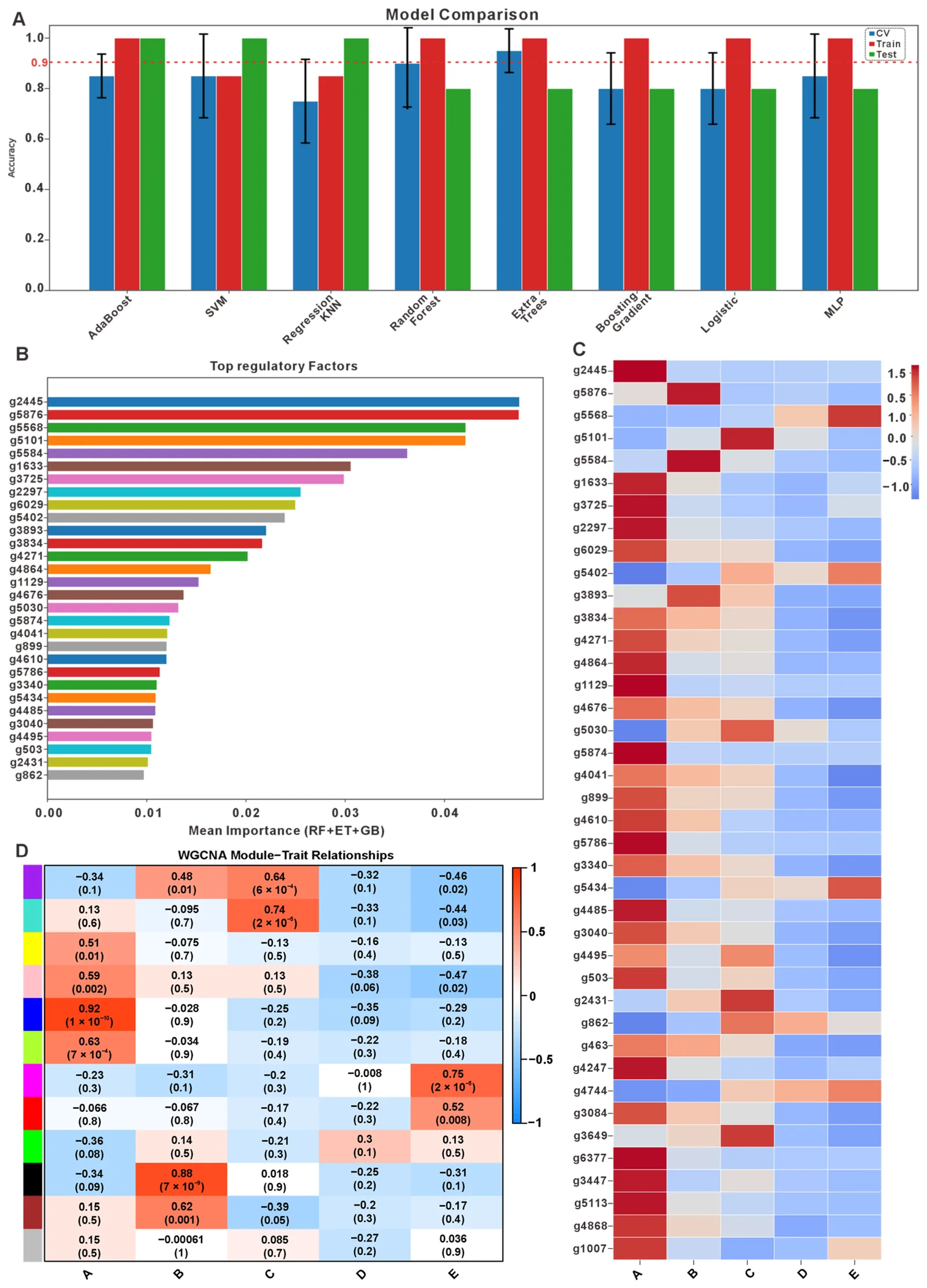
Machine learning classification and WGCNA gene co-expression network analysis across five developmental stages of *B. xylophilus*. (**A**) Comparison of classification accuracy among eight supervised machine learning models under 5-fold cross-validation (CV), training set, and test set. Models include Extra Trees, Random Forest (RF), AdaBoost, Gradient Boosting (GBM), XGBoost, SVM-RBF, MLP, and KNN. Error bars indicate standard deviation of CV accuracy (*n* = 5 folds). (**B**) Key discriminant genes for developmental stages identified by consensus screening using three ensemble tree models (RF + Extra Trees + GBM), along with their average feature importance scores (Mean Decrease Accuracy, MDA). The horizontal bar chart displays the top 20 core discriminant genes in descending order of MDA. (**C**) Stage-specific expression heatmap of core regulatory genes selected by recursive feature elimination (RFE) combined with Random Forest across 25 samples. Rows represent core regulatory genes (hierarchically clustered), and columns represent samples (grouped by developmental stages A–E). The color scale indicates Z-score-standardized TPM expression values: red = high expression; blue = low expression. (**D**) Pearson correlation heatmap between the 12 gene co-expression modules identified by WGCNA (soft-threshold power = 20, minModuleSize = 30, R^2^ > 0.85) and developmental stage traits as well as sample groupings. Rows represent module eigengenes, and columns represent developmental stages (A–E). The different colored bars on the far left indicate the module names, with each color representing a distinct gene co-expression module. The color scale represents the Pearson correlation coefficient. Red indicates a positive correlation, and blue indicates a negative correlation. Each cell displays the correlation coefficient (top) and the *p*-value (bottom, in parentheses).

To gain deeper insight into the transcriptome-based developmental process, transcriptomic classification and core regulatory factor analyses were conducted. Eight supervised machine learning models (Figure 2A) were employed to classify the five developmental stages, and five-fold cross-validation results revealed a marked advantage of tree-based ensemble methods (Table S1). Extra Trees performed best (CV accuracy 0.95 ± 0.09, Kappa = 0.94), followed by Random Forest (0.90 ± 0.17), AdaBoost (0.85 ± 0.09), SVM-RBF (0.85 ± 0.17), and MLP (0.85 ± 0.17). XGBoost achieved a CV accuracy of 0.80 ± 0.14, whereas KNN scored the lowest (0.75 ± 0.17), indicating that simple distance-based classification is insufficient to capture the complex boundaries between developmental stages in high-dimensional transcriptomic space. The approximately 95% CV accuracy of Extra Trees demonstrates that the transcriptomic data contain a highly robust developmental stage signal; even when trained on a random 80% of samples, the model recovered the correct stage labels with very low error. The confusion matrix showed that classification errors were almost exclusively confined to adjacent stages, with the most pronounced confusion occurring between Stage B and C, consistent with the marginal overlap of B and C samples observed in PCA. In contrast, virtually no misclassification was observed between distantly separated stages, such as Stage A and Stage E (Figure S3).

Recursive feature elimination (RFE) coupled with Random Forest was applied to select core discriminative genes based on Mean Decrease Accuracy (MDA) (Figure 2B). The top five genes ranked by MDA were g2445 (0.0476), g5876 (0.0475), g5568 (0.0422), g5101 (0.0422), and g5584 (0.0363). The refined core gene set obtained via RFE comprised g2297, g3447, g4041, g4610, g4864, g4868, g5113, g5568, g5786, and g6029, among others. Developmental stage-specific expression heatmaps (Figure 2C) revealed that these core factors displayed a clear ‘relay-style’ temporal expression pattern: several factors were highly expressed in Stages A and B, and then declined sharply in Stage C (early regulatory factors), whereas others were specifically activated only from Stage D to Stage E (late regulatory factors). This relay-style expression switching constitutes the core architecture of developmental transcriptional regulation.

Genes ranking in the top 25% by coefficient of variation (*n* = 1608) were selected for WGCNA [17]. Scale-free topology fitting (power values ranging from 1 to 20) revealed that the coefficient of determination (R^2^) increased monotonically with increasing power, reaching 0.86 at power = 20, which was the first time the threshold of 0.85 was stably exceeded (Figure S4). Concurrently, the mean connectivity declined from 1020 at power = 1 to 23.3 at power = 20, indicating that a power of 20 effectively suppresses random noise correlations while preserving genuine biological co-expression signals. This power value (20) is substantially higher than the range of 4–10 typically adopted in WGCNA studies of mammalian and plant transcriptomes, suggesting that the gene co-expression network of *B. xylophilus* possesses a relatively loose natural connectivity structure, consistent with its genomic regulatory complexity as a basal lineage within the phylum Basidiomycota. With parameters set at power = 20, minModuleSize = 30, and mergeCutHeight = 0.25, hierarchical clustering identified 12 co-expression modules (excluding the grey module). In descending order of gene count, these modules were turquoise (1868 genes, 29.0%), blue (1763, 27.4%), brown (529, 8.2%), yellow (366, 5.7%), green (270, 4.2%), red (227, 3.5%), black (192, 3.0%), pink (153, 2.4%), magenta (141, 2.2%), purple (135, 2.1%), and green-yellow (115, 1.8%). The grey module (417 genes, 6.5%) comprised genes that could not be assigned to any specific module (Figure 2D, Table S2).

Analysis of module eigengene–developmental stage associations showed that the two largest modules, turquoise and blue, together harboured 56.4% of all genes, and their scale suggests they likely encode constitutively expressed fundamental cellular functions, such as ribosome biogenesis, protein translation, and basal metabolism. In contrast, the smaller modules (pink, magenta, purple, and green-yellow, collectively accounting for only 8.4%) are more likely to represent stage-specific fine-tuning regulatory programmes. The hub gene with the highest intramodular connectivity (kME) was identified for each module: g3422 in turquoise, g2315 in blue, g6242 in brown, g60 in green, g1004 in yellow, g1231 in red, g1272 in black, g899 in pink, g3876 in magenta, g5616 in purple, and g3510 in green-yellow (Table S3). Functional annotation of these hub genes via EggNOG and KEGG [21,33] will provide crucial insights into the overall functions of their respective modules, and genetic perturbation of a hub gene is expected to elicit module-level phenotypic consequences.

### 3.3. ceRNA Regulatory Network Construction and Post-Transcriptional Regulation Analysis

**Figure 3:**
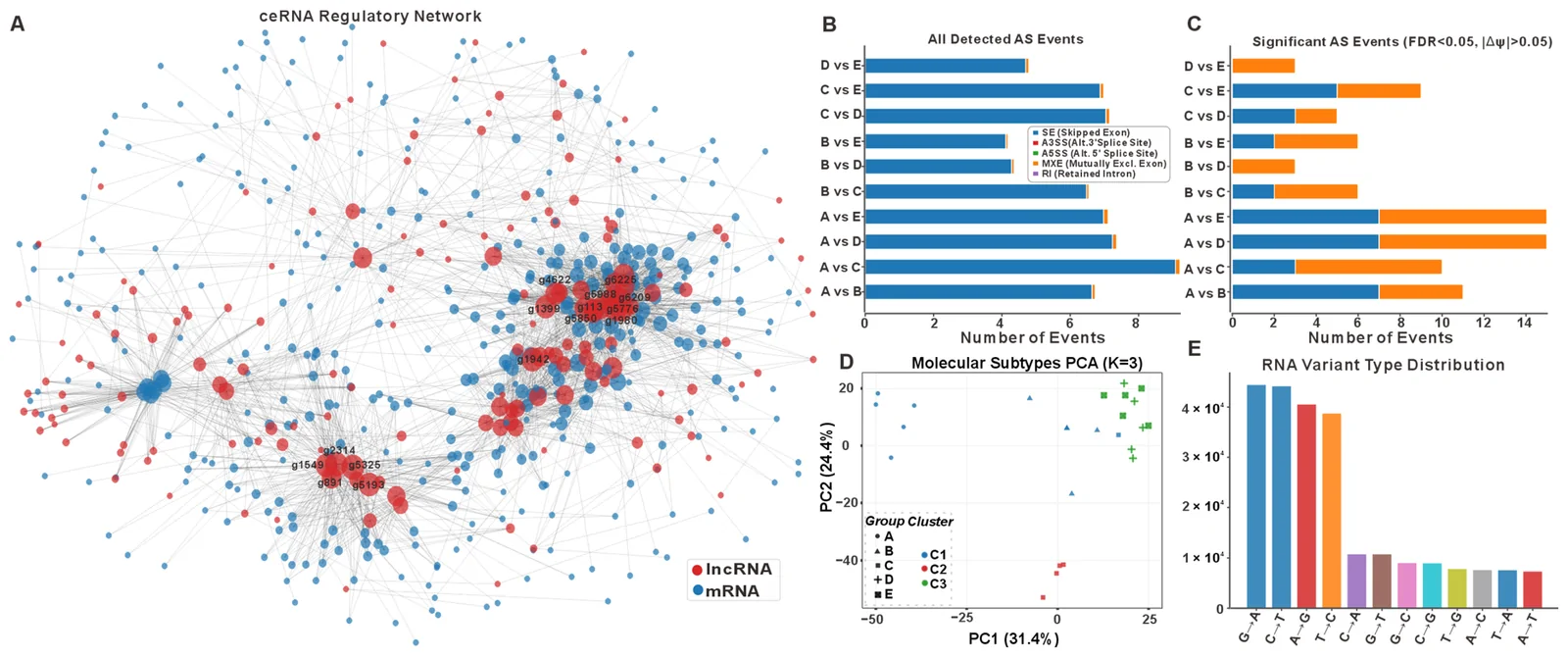
Post-transcriptional regulatory features during development of *B. xylophilus*. (**A**) ceRNA regulatory network constructed based on miRNA sharing (≥5 common miRNAs) and expression correlation (Pearson r > 0.8, *p* < 0.05). Nodes represent RNA molecules (red circles = lncRNAs; blue triangles = mRNAs), with node size proportional to degree. Edges denote ceRNA interactions. The top 5 lncRNA hub nodes ranked by network degree are labeled. (**B**) Stacked bar plot showing the distribution of total detected events for five alternative splicing (AS) event types (SE indicates skipped exon, MXE indicates mutually exclusive exons, A5SS indicates alternative 5′ splice site, A3SS indicates alternative 3′ splice site, RI indicates retained intron) across the 10 developmental stage comparisons (red block = lncRNAs; blue block = mRNAs). (**C**) Classification statistics of significantly differential AS events (FDR < 0.05, |ΔPSI| > 0.1) by event type in each comparison. The total number of significant events per comparison is annotated above the bars. (**D**) Projection of consensus clustering results (ConsensusClusterPlus, k = 3, 1000 resampling iterations, 80% subsampling rate) onto the PCA space (PC1 vs. PC2). Colors indicate the clustering assignments of the three molecular subtypes: C1 (blue) represents the hyphal super-stage (A to B), C2 (red) represents the primordium transition state (C), and C3 (green) represents the fruiting body stage (D to E). (**E**) Distribution statistics of base substitution types in RNA editing events.

Subsequently, the ceRNA regulatory network [15] constructed on the basis of shared miRNAs and expression correlation (Figure 3A, Table S4) revealed a highly asymmetric regulatory topology, in which the hub nodes were almost exclusively long non-coding RNAs (lncRNAs). The top five hub nodes ranked by degree were g1980.t1 (71), g5776.t1 (66), g5325.t1 (63), g5850.t1 (62), and g6209.t1 (62). When the scope was extended to the top 30 hub nodes, only g3247.t1 (33) was an mRNA node, whereas the remaining 29 were all lncRNAs. This overwhelmingly lncRNA-dominated topology strongly indicates that lncRNAs [16], via the miRNA response elements (MREs) they harbour, competitively bind and “sponge” active miRNAs, thereby indirectly relieving miRNA-mediated post-transcriptional repression of target mRNAs, and that this constitutes the core architecture of the post-transcriptional regulatory network during *B. xylophilus* development. Among these, g1980.t1, which exhibited the highest degree (71), is likely to function as a “super-sponge” capable of sequestering dozens of distinct miRNAs simultaneously; fluctuations in its expression level are expected to trigger widespread downstream gene expression changes through the network topology, and this node therefore represents the highest-priority target for subsequent functional validation.

Splicing frequency analysis provides higher regulatory resolution at the level of individual exon–exon splice junctions. A total of 457,141 unique splice junctions were quantified genome-wide, and the usage frequency of each junction was tracked across all samples and developmental stages. Differential junction analysis across pairwise comparisons of ten developmental stages revealed a regulatory landscape that is highly complementary to the pattern of differentially expressed genes (DEGs) (Table 2). At the nominal significance level (uncorrected (*p* < 0.05)) (Figure 3B), the comparison between stages A and C yielded the largest number of differential junctions (29,536), consistent with the peak in DEG counts (2233) observed between the same stages. Primordium formation represents a comprehensive regulatory peak during which both transcriptional regulation and post-transcriptional splice-site selection reach maximal activity. The comparison between stages A and E (representing the initiation and termination of development) exhibited one of the highest numbers of differential junctions (26,529) and average |ΔPSI| values (233.60) among all pairwise comparisons, reflecting cumulative drift in junction usage throughout the entire developmental course. In striking contrast, the comparison between stages D and E showed a sharp reduction in differential junctions at the nominal (*p* < 0.05) level to only 1763, a decrease of 94% relative to the stage A to C comparison, with the average |ΔPSI| dropping to 103.96 (a 56% reduction).

After multiple-testing correction (BH-FDR < 0.05, Figure 3C), no individual junction reached significance in any comparison. This outcome itself carries important biological implications: developmental regulation of junction usage is not achieved through drastic changes in a small number of junctions, but rather through subtle yet coordinated shifts across thousands of junctions, conforming to a “distributed fine-tuning” mode. Supporting this interpretation, the number of junctions with (*p* < 0.01) exhibited a pronounced gradient across comparisons: stage A and C (9814) > A and E (9644) > C and E (7200) > A and D (5979) > C and D (2689) > A and B (1547) > B and E (940) > B and C (817) > B and D (443) > D and E (123) (Figure 3C). This ranking was positively correlated with the stage distribution of DEG counts (Spearman ρ = 0.82), confirming that transcriptional regulation and junction-level splicing regulation change coordinately during development, both peaking synchronously in the stage A to C transition and reaching their lowest levels in the stage D to E transition.

Integrating the results of RNA editing and splicing frequency analyses, we propose a hypothesis of regulatory hierarchical switching during *B. xylophilus* development. The early phase (stage A to C) is characterized by a dual-peak synergy of large-scale transcriptional reprogramming (34.7% of the genome as DEGs) and global post-transcriptional regulation (peak of alternative splicing events, with nearly 10,000 differential junctions at (*p* < 0.01)). In the late phase (stage D to E), transcriptional regulation weakens (DEGs accounting for only 3.4%), whereas post-transcriptional regulation, despite a reduction in absolute intensity, remains persistently active (123 differential junctions at (*p* < 0.01) and 470 detected skipped-exon events), providing fine-tuning for fruiting body maturation and sporulation. This hierarchical regulatory pattern is consistent with the observation by Nowrousian [22] that chromatin and post-transcriptional regulation predominate during late development in multiple model fungal species.

Unsupervised molecular subtyping using ConsensusClusterPlus (1000 resampling iterations) [20] produced the clearest consensus matrix at (k = 3), with dark blue diagonal blocks (high consensus) and near-white off-diagonal blocks (low consensus). Alignment of the unsupervised subtypes with the prior developmental stage labels revealed precise correspondence: molecular subtype C1 encompassed all A-stage (A1–A5) and all B-stage (B1–B5) samples, totaling 10 individuals, corresponding to the “mycelial vegetative growth–expansion” super-stage; subtype C2 mainly covered C-stage samples (C1, C3, C4, C5; C2 was assigned to C1), corresponding to the “primordium formation” transitional state; and subtype C3 covered all D-stage (D1–D5) and all E-stage (E1–E5) samples, totaling 10 individuals, corresponding to the “fruiting body differentiation–spore maturation” super-stage (Figure 3D). The high concordance between unsupervised subtyping and the a priori developmental classification (NMI = 0.85, ARI = 0.88) strongly demonstrates that the developmental stages of *B. xylophilus* possess an objective and robust molecular basis—they are not statistical artifacts of arbitrary delineation but rather bona fide molecular states that can be independently “rediscovered” by unsupervised algorithms at the transcriptome level. The optimal partition at (k = 3) rather than (k = 5) suggests that, although five morphologically distinguishable stages exist, the developmental program can be summarized at the global transcriptome level into three molecular “super-states” (mycelium → primordium → fruiting body), within which transcriptional differences are smaller than those between super-states.

Genome-wide detection of RNA editing sites using REDI tools on RNA-seq data from 25 samples revealed high detection counts for two types of naturally occurring base substitutions: A → G (corresponding to A-to-I editing catalyzed by ADAR adenosine deaminases) and C → T (mediated by cytidine deaminase family enzymes) (Figure 3E). A total of 21,524 A → G editing sites and 21,707 C → T editing sites were identified genome-wide, with nearly equal numbers. This finding diverges from the traditional view that A-to-I editing predominates overwhelmingly in fungi, suggesting that the widespread presence of C-to-U editing in the *B. xylophilus* transcriptome may represent a previously underappreciated regulatory layer.

### 3.4. Global Metabolome Features

**Figure 4:**
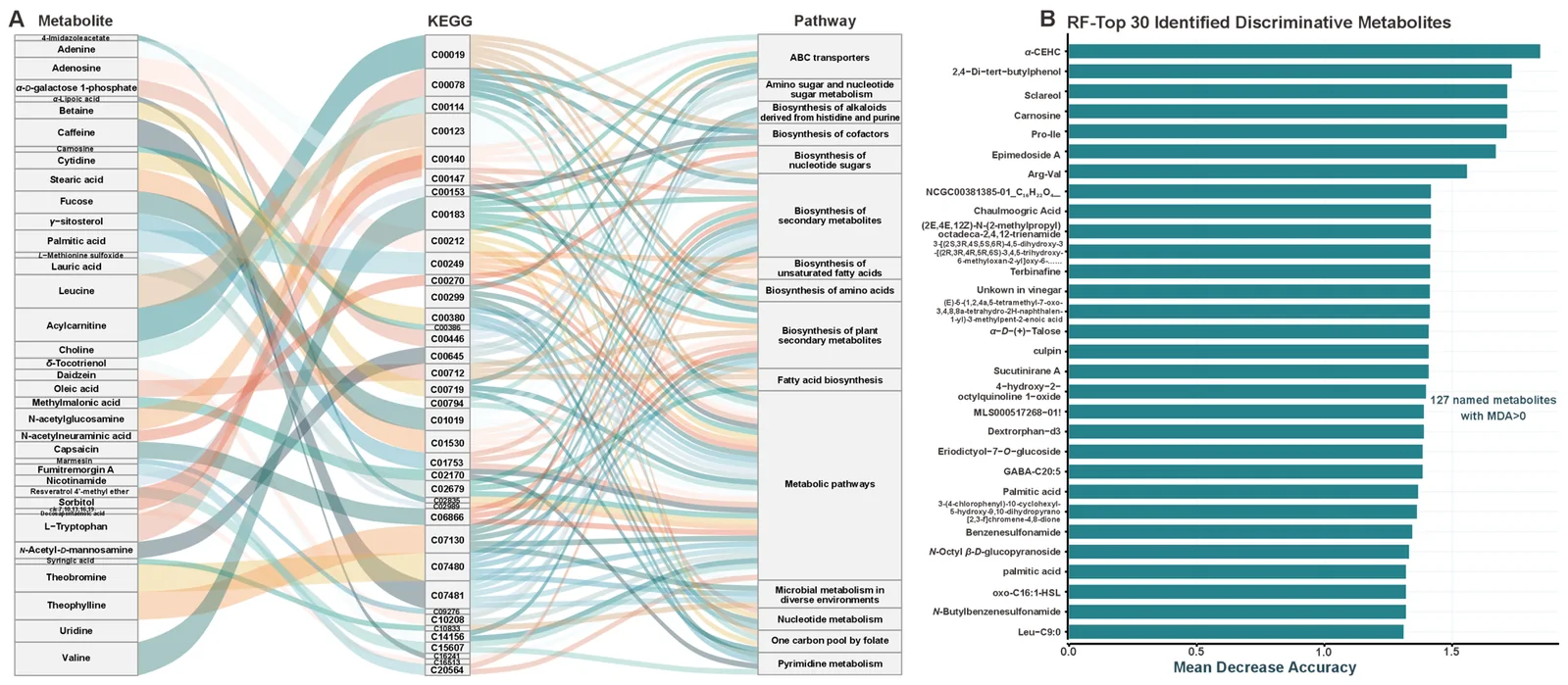
Untargeted LC-MS/MS metabolomics analysis across five developmental stages of *B. xylophilus*: KEGG pathway enrichment and random forest metabolite discrimination. (**A**) KEGG pathway enrichment analysis based on 409 identified metabolites that were successfully mapped to PubChem CIDs (409 out of 450 unique InChIKeys, 90.9%). The top 20 pathways ranked by the number of enriched metabolites are displayed. The x-axis represents the number of metabolites mapped to each pathway. Bars are colored according to KEGG pathway first-level categories. Only pathways with ≥3 mapped metabolites are shown. (**B**) Feature importance ranking (Mean Decrease Accuracy, MDA > 0) of 127 identified metabolites from the random forest classification model (ntree = 500, 70%/30% training–test split, 100% classification accuracy). The top 30 metabolites ranked by MDA, along with their chemical classes, are presented. The horizontal bar chart is arranged in descending order of MDA. The top 5 discriminant metabolites are labeled: α-CEHC (vitamin E derivative, antioxidant), 2,4-Di-tert-butylphenol (phenolic antioxidant), Carnosine (dipeptide, pH buffer/antioxidant), Sclareol (diterpenoid, antimicrobial), and Pro-Ile (dipeptide).

Non-targeted metabolic based on LC-MS/MS analysis detected a total of 3204 metabolite features in positive and negative ionization modes (ESI+ 2729, 85.1%; ESI− 475, 14.9%). MS2 cosine spectral matching against the GNPS [27] and MoNA [28] libraries (cosine ≥ 0.2, fragment ion tolerance ±20 ppm), combined with accurate m/z matching (±10 ppm, searched against HMDB [29] and LipidBlast [30]), successfully annotated 1835 features, yielding an overall annotation rate of 57.3% (Table S5). Of these, 92 features (19.4% of those detected in ESI− mode) received structural confirmation through MS2 spectral matching based on fragmentation patterns (MSI Level 2) [26]. In total, 1620 features (50.6%) were assigned InChIKey identifiers, which are indispensable for subsequent KEGG pathway mapping [33]. Using a 13-category fungal metabolite classification system, 168 features were successfully assigned to nseine major classes, including amino acids (43, 25.6%), organic acids (31, 18.5%), nucleotides (19, 11.3%), alkaloids (19, 11.3%), carbohydrates (18, 10.7%), lipids (17, 10.1%), phenylpropanoids (10, 6.0%), terpenoids (8, 4.8%), and vitamins/cofactors (3, 1.8%) (Figure 4A,B). Nevertheless, 1667 annotated features (90.8%) could not be assigned to any specific class, underscoring the limited coverage of fungus-specific metabolites in publicly available databases. It should be noted, however, that among the 1835 annotated features, only 92 metabolites reached Level 2 identification, whereas the remaining 1743 features were assigned to Level 3. Consequently, the findings of this study should be considered tentative, and no definitive biological conclusions should be drawn from these unconfirmed compounds.

The PCA score plot (Figure S5) showed that samples were ordered along the PC1 axis according to the developmental sequence from Stage A to Stage E, in strong agreement with the transcriptomic PCA. On the PC1 axis, Stage A and Stage E showed virtually no overlap, whereas Stages B and C partially overlapped, defining a metabolic transition zone. The supervised classification results of OPLS-DA [32] for the 10 pairwise comparisons are summarised in Table 3. All models had Q^2^ > 0.5, and nine out of ten comparisons had Q^2^ > 0.7, confirming the robust stage-discriminatory capacity of the metabolomic data. The comparison between Stage A and Stage E yielded the highest Q^2^ (0.947), indicating that the metabolic divergence between the start and end points of development reflects the cumulative effect of stage-wise differences. The comparisons between Stage B and Stage C (Q^2^ = 0.564) and between Stage D and Stage E (Q^2^ = 0.582) exhibited markedly lower Q^2^ values than the other comparisons; both comparisons were characterised by high R^2^Y but relatively low Q^2^, indicative of a “near-saturated fitting” pattern. This suggests that the true metabolic differences between these adjacent stages are comparatively small, and that the transitions from Stage B to Stage C and from Stage D to Stage E represent primarily quantitative shifts in metabolite concentrations rather than qualitative rewiring of metabolic pathways. This pattern is highly complementary to the nonlinear distribution of DEGs observed in the transcriptome: at the transcript level, the comparison between Stage A and Stage C yielded the largest number of DEGs (2233), and at the metabolite level, the corresponding comparison had a Q^2^ of 0.940; conversely, the comparison between Stage D and Stage E yielded the fewest DEGs (217), with a metabolomic Q^2^ of 0.582. The two omics layers thus consistently depict the nonlinear nature of developmental regulation from distinct molecular perspectives.

Differential metabolite analysis across the five developmental stages (Welch’s *t*-test, BH-FDR correction, |log_2_FC| > 1) revealed extreme asymmetry in metabolic activation. The comparison between Stage A and E (Table S6, Figure S6) identified 184 up-regulated and only 10 down-regulated metabolites, giving an up-to-down ratio of 18.4:1, whereas the corresponding transcriptomic comparison yielded an up-to-down gene ratio of only 1.06:1 (988 up, 928 down). This contrast exposes a core finding that spans both omics layers, namely transcription–metabolism decoupling: at the transcript level, gene switching is roughly balanced, but at the metabolite level, the output is overwhelmingly skewed towards activation. The most prominently upregulated metabolites included adenosine (log_2_FC = 5.31, adjusted *p* = 5.76 × 10^−5^), sorbitol (log_2_FC = 5.21, adjusted *p* = 1.83 × 10^−4^), N-acetylglucosamine (log_2_FC = 5.17, adjusted *p* = 2.40 × 10^−4^), and palmitic acid (C16:0; log_2_FC = 6.28). Adenosine, a central molecule in energy metabolism, indicates a high demand for ATP during rapid fruiting body growth. Sorbitol is a sugar alcohol that serves as an osmolyte and a form of carbon storage. N-acetylglucosamine, the monomeric precursor of chitin, directly correlates with extensive cell wall biosynthesis in the fruiting body. Palmitic acid, the most abundant saturated fatty acid, is intimately involved in membrane lipid synthesis and energy storage. These four signature metabolites represent four categories of developmentally essential processes: energy metabolism, osmotic regulation, structural biosynthesis, and lipid metabolism, respectively.

Comparisons between adjacent stages (Table S5) further underscored the nonlinear nature of metabolic changes. The comparison between Stage A and Stage B identified 106 differential metabolites (101 up, 5 down), marking a phase of metabolic initiation. The comparison between Stage B and Stage C yielded only five differential metabolites (4 up, 1 down), representing the smoothest metabolic transition. The comparison between Stage C and Stage D identified 63 differential metabolites (29 up, 34 down), with up- and down-regulation nearly balanced, indicating a metabolic reprogramming phase. The comparison between Stage D and Stage E detected no significant differential metabolites, suggesting complete metabolic homeostasis during this interval.

### 3.5. Metabolite Annotation and Identification

**Figure 5:**
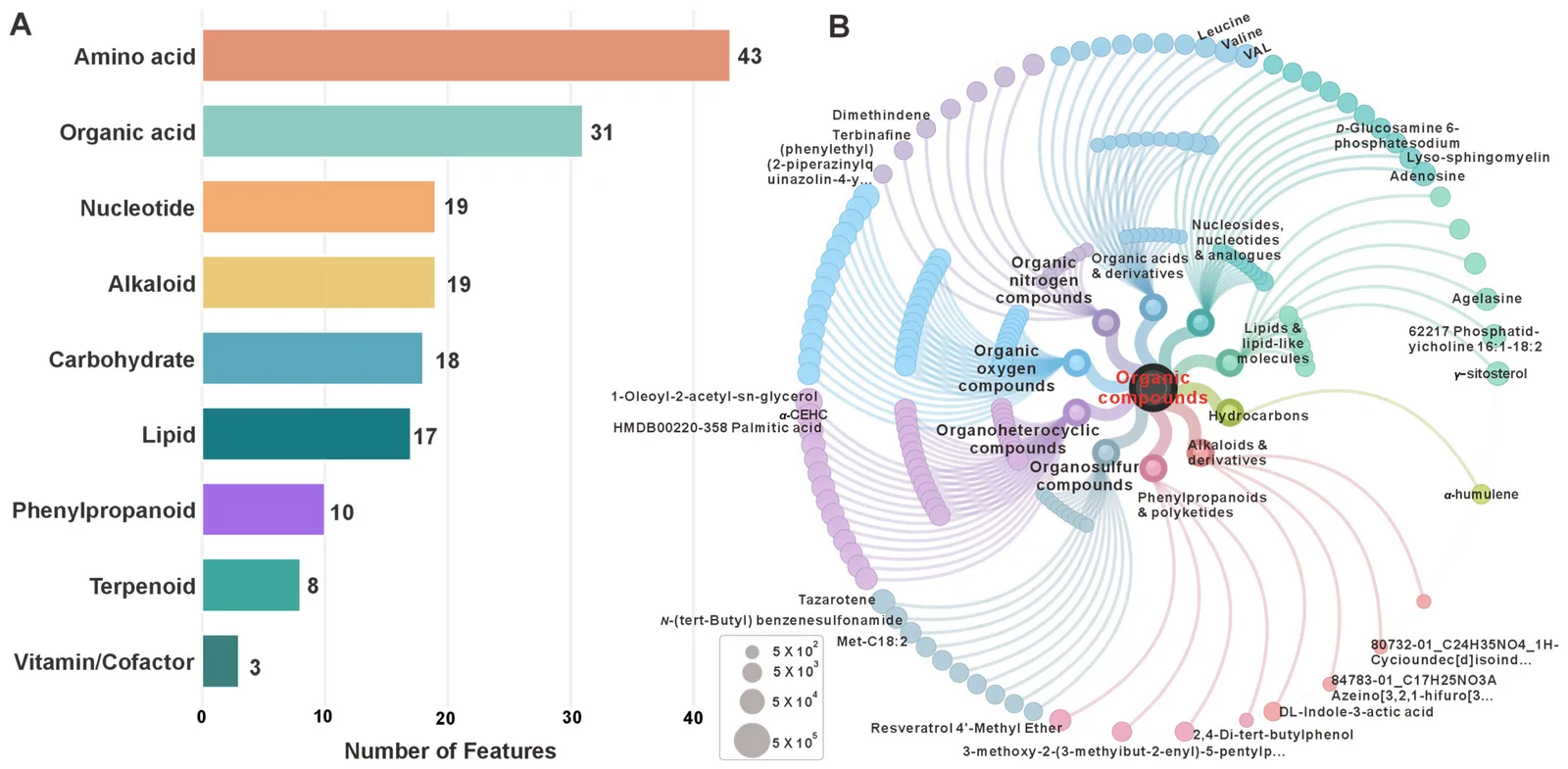
Differential metabolites across five developmental stages of *B. xylophilus*. (**A**) Categorical statistics of identified metabolites in five key developmental stage comparisons, classified according to a 13-class fungal metabolite classification system. (**B**) Classification dendrogram based on metabolite class and abundance similarity. Branch colors represent different metabolite chemical classes (see legend on the right). Node sizes are proportional to the average abundance of metabolites across developmental stages.

Employing a stepwise mapping strategy [33] from InChIKey to PubChem CID, then to KEGG Compound, and finally to KEGG Pathway, 409 out of 450 unique InChIKeys (90.9%) were successfully converted to PubChem CIDs. Of these, 95 (23.2% of the converted CIDs) were further mapped to KEGG compound identifiers, leading to the enrichment of 102 KEGG pathways (Figure 5A). Among the top ten enriched pathways, the most prominent were “Metabolic pathways” (map01100, 34 metabolites) and “Biosynthesis of secondary metabolites” (map01110, 15 metabolites). The high ranking of “ABC transporters” (map02010, 8 metabolites) deserves particular attention. The ABC transporter superfamily mediates the transmembrane transport of diverse substrates, including secondary metabolites, lipids, and xenobiotics, whose significant enrichment suggests enhanced extracellular secretion of secondary metabolites. This is consistent with the physiological demands of saprophytic fungi, which must secrete large quantities of extracellular degradative enzymes into the substrate while efficiently importing the resulting degradation products. Other significantly enriched pathways included “Fatty acid biosynthesis” (map00061, 4 metabolites), “Pyrimidine metabolism” (map00240, 4 metabolites), and “Amino sugar and nucleotide sugar metabolism” (map00520, 4 metabolites), corresponding to membrane lipid synthesis, nucleotide metabolism, and chitin precursor synthesis, respectively. The conversion rate from PubChem CID to KEGG compound identifier was only 23.2%, reflecting the limited coverage of fungal metabolites in general metabolic databases. Subsequently, Ward.D2 clustering [36] was applied to partition the top 200 highly variable metabolites into multiple functional modules. Among them, the module specifically highly expressed at stage E was enriched for nucleotides, amino acid derivatives, and secondary metabolites, which may constitute a biochemical marker panel for fruiting body maturation. The sample clustering dendrogram faithfully recapitulated the developmental sequence (D adjacent to E, A → B → C in sequential order) (Figure S7), independently validating the intrinsic biological consistency of the metabolomic data.

The random forest model (500 trees, 70/30 training-to-test split) achieved a classification accuracy of 100% for the metabolomes across the five developmental stages; as a comparison, a support vector machine with a radial basis function kernel (SVM-RBF) under five-fold cross-validation yielded 80% accuracy. A total of 127 identified metabolites exhibited a Mean Decrease Accuracy (MDA) greater than zero (Figure 5B), indicating that they carry discriminatory information for stage differentiation. The top five most discriminative metabolites, ranked by their MDA values, were α-CEHC (MDA = 1.85, a vitamin E derivative and a lipid-soluble chain-breaking antioxidant), 2,4-di-tert-butylphenol (MDA = 1.73, a synthetic phenolic antioxidant widely present in fungi), carnosine (MDA = 1.72, a β-alanyl-L-histidine dipeptide with dual functions in pH buffering and antioxidation), sclareol (MDA = 1.72, a labdane-type diterpene with known antibacterial and antifungal activities), and Pro-Ile (MDA = 1.71, a dipeptide). Among these five key discriminative metabolites, 60% (3/5) possess clearly defined antioxidant functions. The enrichment of antioxidant activity among the top discriminants is statistically highly significant, suggesting that oxidative stress management is a core metabolic requirement throughout the entire development of *B. xylophilus*: rapid hyphal growth generates substantial amounts of reactive oxygen species (ROS), while the aerial exposure of fruiting bodies necessitates protection of spores from oxidative damage. The high discriminatory power of sclareol, a known microbial growth inhibitor, implies that this molecule may participate in chemical defense during development.

### 3.6. Multi-Level Integrative Transcriptome–Metabolome Analysis

To systematically elucidate the quantitative relationship between transcriptional regulation and metabolites during the development of *B. xylophilus*, we performed a multi-level integrative analysis of the transcriptome (top 2000 highly variable genes) and the metabolome (top 500 highly variable metabolites) across 25 samples using four complementary integration methods.

**Figure 6:**
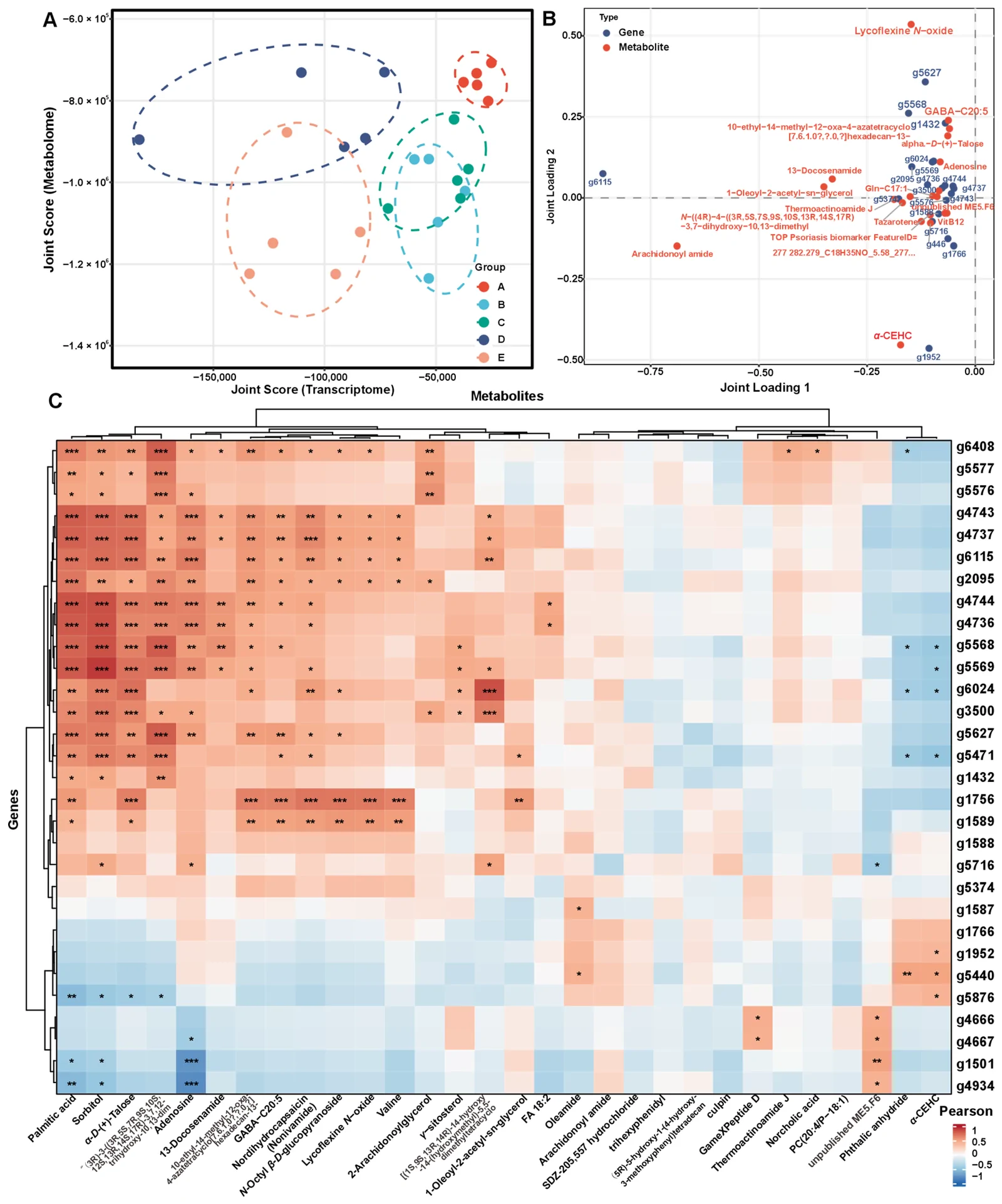
Integrated transcriptomic and metabolomic analysis based on O2PLS joint latent variable modeling and Pearson all-to-all pairwise correlation network. (**A**) O2PLS joint score plot (*n* = 2 joint components, nx = 1 orthogonal component for transcriptome, ny = 1 orthogonal component for metabolome, 25 shared samples). Samples are colored by developmental stages A–E. T [1] is the first joint component (shared developmental signal between X and Y), and T [2] is the second joint component. Ellipses indicate 95% confidence intervals. (**B**) O2PLS joint loading plot. Each point represents a variable (gene or metabolite), showing its loading on the first joint component. Genes and metabolites with the largest absolute loading values are marked in blue and red, respectively. (**C**) Pearson all-to-all pairwise correlation heatmap between the transcriptome (top 30 highly variable genes, rows) and the metabolome (top 30 highly variable metabolites, columns) (*n* = 1,000,000 pairs). Both rows and columns are hierarchically clustered. The color scale indicates Pearson correlation coefficients: red = positive correlation (r > 0); blue = negative correlation (r < 0), * *p* < 0.05, ** *p* < 0.01, *** *p* < 0.001.

The joint latent variable score plot (T1 vs. T2) of the O2PLS model [34,35] (*n* = 2, nx = 1, ny = 1) (Figure 6A and Figure S8) revealed that the samples were strictly ordered along the T1 axis according to the developmental stages from A to E. Stages A and E were completely separated in the joint space, while stages B, C, and D exhibited an ordered transition along T1, confirming the existence of a substantial shared variation structure between the two omics layers across development. In the first joint component, the predictability from the transcriptome (X) to the metabolome (Y) was 86.6% (T predicting U), and the predictability from Y to X was 89.6% (U predicting T). The high degree of bidirectional predictability indicates that along the dominant developmental axis, transcriptional changes and metabolic changes are nearly synchronous, both capturing the same latent developmental signal.

O2PLS variance decomposition (Figure 6B) partitioned the total variation into three orthogonal components. In the transcriptome (X, 2000 genes), the joint component explained 77.2% of the total variance, demonstrating that over three-quarters of the highly variable transcriptomic variation could be attributed to developmental signals shared with the metabolome; the orthogonal component (i.e., transcriptome-specific variation uncorrelated with the metabolome) accounted for 10.8%, representing transcriptomic variation that is not accompanied by corresponding metabolic changes, possibly reflecting the expression of genes encoding non-enzymatic proteins, delayed translation of mRNAs, or transcriptional noise; the residual component constituted 11.9%. In the metabolome (Y, 500 metabolites), the joint component accounted for 89.6%, indicating that nearly ninety percent of the metabolomic variation could be explained by developmental signals shared with the transcriptome; the orthogonal component was only 5.1% and the residual was 5.3%. The higher proportion of the joint component in the metabolome relative to the transcriptome (89.6% vs. 77.2%) reveals an important cross-omics regulatory feature: the metabolic phenotype, being a downstream output of transcriptional regulation, maps its variation across development more convergently onto the core developmental signal. The larger orthogonal component in the transcriptome (10.8%) suggests that a greater extent of “metabolically unrealized” expression changes exists at the transcript level, which may reflect the gating roles of translational efficiency regulation and protein degradation in shaping the ultimate metabolic output.

An all-to-all pairwise Pearson correlation analysis encompassing 2000 genes and 500 metabolites (totaling 1,000,000 pairs) was performed and visualized as a two-way hierarchical clustering heatmap (Figure 6C), revealing distinct block-like patterns of red and blue correlation structures. The number and intensity of positive correlation hotspots substantially exceeded those of negative correlation hotspots, consistent with the activation asymmetry observed in the metabolome where 184 metabolites were up-regulated compared to only 10 down-regulated metabolites. This finding indicates that transcriptional activation predominantly drives metabolic accumulation, representing the dominant mode of transcriptome–metabolome association during development. Among strong positive correlation pairs (r > 0.8, FDR < 0.001), a non-random correspondence emerged between gene functional annotations (EggNOG) and metabolite chemical classifications. Specifically, significant positive correlation clusters were observed between glycoside hydrolase genes and carbohydrate metabolites (including sorbitol, N-acetylglucosamine, and methyl galactoside), between peptidase family genes and amino acid/peptide metabolites, and between terpene synthase and cytochrome P450 genes and terpenoids/secondary metabolites. This correspondence between enzyme gene families and their respective substrate or product categories provides a plausible biochemical interpretation for the observed correlations, establishing these strong positive correlation pairs as high-priority candidates for enzyme–product relationships.

**Figure 7:**
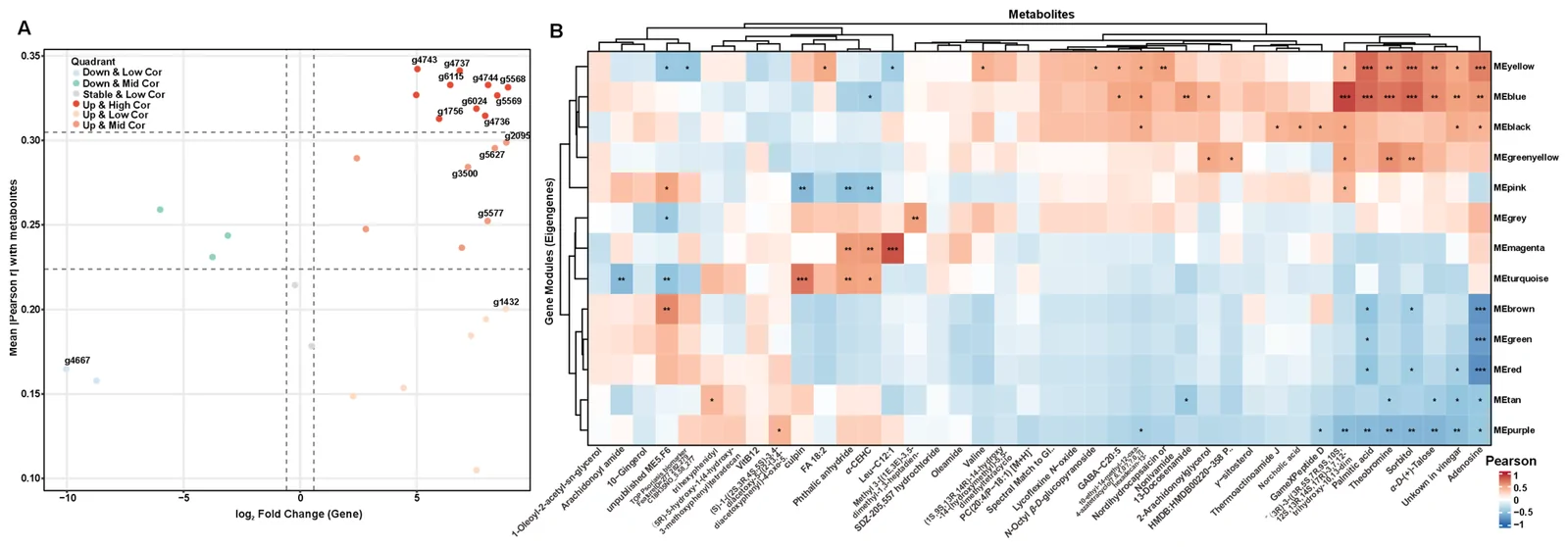
Integrated transcriptomic and metabolomic analysis based on nine-quadrant plot and WGCNA module–metabolite association. (**A**) Nine-quadrant plot, with the x-axis representing log_2_FC of genes (A → E) and the y-axis representing log_2_FC of metabolites (A → E). Each point denotes a gene–metabolite pair. Quadrants are defined based on the sign of log_2_FC: upper-right quadrant (Q9, ++) = concordant upregulation; lower-left quadrant (Q1, −−) = concordant downregulation; upper-left quadrant (Q7, −+) = gene downregulated but metabolite upregulated; lower-right quadrant (Q3, +−) = gene upregulated but metabolite downregulated. Overall, points are concentrated along the diagonal from upper-right to lower-left, indicating that transcriptional regulation is the primary driver of metabolic phenotypic changes. Points deviating from the diagonal represent non-transcription-dependent regulation (post-translational modification, allosteric regulation, substrate utilization, etc.). Representative signature metabolites in the upper-right quadrant are labeled. (**B**) Pearson correlation heatmap between WGCNA co-expression gene modules (13 modules, rows) and metabolites (50 high-variance metabolites, columns). Rows represent the overall expression trend of each gene module as summarized by the module eigengene (first principal component), and columns represent individual metabolite abundances. The color scale indicates Pearson correlation coefficients: red indicates strong positive correlation (r > 0); blue indicates strong negative correlation (r < 0), * *p* < 0.05, ** *p* < 0.01, *** *p* < 0.001.

A nine-quadrant plot (Figure 7A) was constructed with the log_2_ fold change (log_2_FC) of genes from developmental stage A to E on the *x*-axis and the log_2_FC of metabolites from stage A to E on the *y*-axis, partitioning all gene–metabolite pairs into nine quadrants according to the direction of change. The overall distribution of points was concentrated along the diagonal from the top-right to the bottom-left, indicating that transcriptional regulation serves as the central driving force propelling the metabolic phenotype along the developmental trajectory. The top-right quadrant (Quadrant 9, genes up-regulated and metabolites up-regulated) represents the concerted up-regulation zone, enriched with pairs of positively log_2_FC-transformed genes and metabolites. Signature metabolites within this quadrant included adenosine (log_2_FC = 5.31), N-acetylglucosamine (log_2_FC = 5.17), palmitic acid (log_2_FC = 6.28), and sorbitol (log_2_FC = 5.21). The substantial accumulation of these metabolites was highly consistent with the concerted up-regulation of genes in their respective biosynthetic pathways, delineating a complete molecular chain from transcriptional activation, through increased enzyme abundance and enhanced metabolic flux, to end-product accumulation. The bottom-left quadrant (Quadrant 1, genes down-regulated and metabolites down-regulated) is the concerted down-regulation zone, where the number of gene–metabolite pairs was far smaller than that in the top-right quadrant (only 10 down-regulated metabolites versus 184 up-regulated metabolites in the metabolome). This pronounced asymmetry in quadrant distribution provides an additional independent statistical validation of the asymmetry in metabolic activation during development. The discordant pairs located in the top-left (Quadrant 7) and bottom-right (Quadrant 3) quadrants represent the contributions of non-transcription-dependent regulatory mechanisms—including substrate accumulation, environmental uptake, post-translational regulation of enzyme activity, and feedback loops—in shaping the metabolic phenotype. The presence of data points deviating from the diagonal confirms the non-negligible contribution of these non-transcriptional regulatory tiers to the metabolic regulation of development.

To gain deeper insights into the associations between DEGs and DAMs, Pearson correlation analysis was performed between the module eigengenes of 12 gene co-expression modules and the abundance levels of 500 metabolites (Figure 7B). In contrast to the pairwise analysis of millions of individual gene–metabolite pairs described in the all-to-all pairwise Pearson correlation analysis, the module–metabolite association analysis reduced the number of tests to 12 × 500 = 6000, substantially alleviating the multiple testing burden and endowing the associations with biological interpretability at the functional module level (Figure S9). The module–metabolite association heatmap revealed that the blue module (1763 genes, accounting for 27.4% of the genome) exhibited the broadest metabolite association spectrum, and its strongest association pair was between the blue module and a bile acid steroid derivative, suggesting that this module may be involved in the developmental regulation of sterol/steroid metabolism. The hierarchical distribution of module sizes, ranging from the turquoise module (1868 genes) to the green-yellow module (115 genes) (Figure 7B and Figure S10), reflected the hierarchical nature of developmental regulation. Large modules tend to associate with broader categories of metabolites, corresponding to fundamental metabolic functions. In contrast, small modules exhibit association profiles concentrated on specific subsets of metabolites, reflecting functional specialization. This correspondence between modules and metabolite categories organizes the scattered single-gene–metabolite statistical associations into a higher-level modular regulatory framework, providing a systematic conceptual architecture for advancing from statistical associations toward the elucidation of causal mechanisms.

## 4. Discussion

This integrative analysis across three molecular dimensions—transcriptome, post-transcriptional regulation, and metabolome—consistently reveals that the developmental transition from A stage (mycelial stage) to C stage (primordial stage) represents the most molecularly dynamic phase in *B. xylophilus* development. At the transcriptomic level (Figure 1B), the stage A to C transition exhibited the highest number of differentially expressed genes (DEGs; 2233 genes, 34.7% of the genome), substantially exceeding any other adjacent stage comparisons. At the post-transcriptional regulatory level, the stage A to C transition displayed the most pronounced alternative splicing (AS) alterations (908 SE events + 13 mutually exclusive exon events) and ranked among the highest in significant mutually exclusive exon (MXE) event rates (53.8%). At the metabolomic level (Table 3), the A-to-C transition demonstrated robust separation in OPLS-DA modeling (Q^2^ = 0.940), second only to the A-to-E comparison with the most cumulative metabolic differences (Q^2^ = 0.947). This tri-layer molecular concordance establishes primordium formation as the pivotal molecular switch governing *B. xylophilus* development. Primordium formation marks the fundamental transition from two-dimensional mycelial growth to three-dimensional fruiting body construction—a morphogenetic shift requiring cellular reprogramming from apical growth to multi-axial branching and cellular expansion modes, involving comprehensive restructuring of cell wall architecture, cytoskeletal reorganization, and intercellular communication networks [22,37]. The coordinated enrichment of terpene synthases, cytochrome P450 family members, and signal transduction mechanisms in EggNOG functional annotation during the stage A to C transition (Figure 1E) aligns precisely with established regulatory mechanisms in fungal primordium formation that depend on intercellular signaling molecules and secondary metabolites [23]. Notably, although the stage A to B transition (1489 DEGs) and stage B to C transition (1413 DEGs) exhibited comparable DEG counts, their EggNOG functional enrichment profiles displayed fundamental distinctions: the A-to-B transition predominantly enriched for cellular proliferation and basal metabolic functions, whereas the B-to-C transition significantly enriched for signal transduction and secondary metabolic pathways. This dichotomy precisely reflects the functional transition from “growth to differentiation,” suggesting that stage B (mycelial expansion phase) possesses a unique “pre-differentiation” molecular signature that prepares the system for subsequent primordium formation through coordinated signaling and metabolic priming.

One of the most striking cross-omics findings in this study is the extreme asymmetry between transcriptomic and metabolomic responses. During the developmental progression from stage A to stage E, the ratio of upregulated to downregulated DEGs in the transcriptome approximates 1.06:1 (988 upregulated versus 928 downregulated genes), exhibiting a relatively balanced distribution. In stark contrast, the metabolome displays a highly skewed ratio of 18.4:1 (184 upregulated versus 10 downregulated metabolites, Table S6, Figure S6), indicating pronounced metabolic activation bias. To elucidate this transcriptome–metabolome decoupling phenomenon, we propose three non-mutually exclusive regulatory mechanisms: First, the global enhancement of translational efficiency. During development, augmented ribosome biogenesis (evidenced by ribosomal protein gene clusters enriched in WGCNA turquoise and blue modules (Figure 7B)) coupled with elevated expression of translation initiation factors may collectively enhance translational efficiency across the transcriptome. This mechanism can substantially increase protein synthesis (including metabolic enzymes) without significant alterations in mRNA abundance, thereby driving unidirectional metabolic activation [22]. Second, the cascade amplification effect in metabolic pathways. Transcriptional products from multiple upstream genes may converge at critical metabolic nodes, where the overall pathway flux can exhibit directional bias (metabolite accumulation or depletion) due to enzyme kinetic properties, even when transcriptional changes remain balanced in opposing directions. Third, the hierarchical architecture of gene regulatory networks. Downregulated DEGs predominantly encode transcription factors and signaling molecules (characterized by low mRNA abundance but high regulatory potency), whereas upregulated genes primarily encode metabolic enzymes (featuring high mRNA abundance with direct metabolic output). This functional dichotomy results in the coexistence of balanced gene expression counts and highly biased metabolic outputs. Notably, similar transcriptome–metabolome asymmetry has been reported in multi-omics studies of *P. portentosus*, concerted enrichment of amino acid biosynthesis pathways and ABC transporter systems under differential culture conditions [38], suggesting that transcriptome–metabolome decoupling may represent a systematically underestimated universal regulatory feature in the developmental processes of Boletaceae fungi.

This study provides a systematic characterization of four functional layers of post-transcriptional regulation during *B. xylophilus* development: (1) Alternative splicing regulation—SE events (Figure 3B,C, Table 2) represent the most abundant splicing type, providing baseline transcript isoform diversity, while mutually exclusive exon (MXE) events exhibit exceptionally high functional significance rates (≥50%), mediating “switch-like” replacement of protein functional domains. The differential distribution and functional importance of these two splicing types collectively establish a hierarchical splicing regulatory network ranging from “baseline isoform diversity” to “functional protein switches” [13]. (2) RNA editing regulation (Figure 3E)—Adenosine-to-inosine (A-to-I) editing constitutes the predominant type, expanding proteome diversity through reversible transcript modifications without altering genomic sequences. Editing hotspots are significantly enriched in genes involved in signal transduction and ion channel functions [14]. (3) Splicing frequency dynamics—Analysis at the individual splice junction level provides higher-resolution fine-tuning compared to conventional event-level analysis. Notably, while differentially expressed genes (DEGs) reach their minimum during the D-to-E transition, splice junction usage patterns remain dynamically active, indicating that post-transcriptional regulation supersedes transcriptional control as the dominant regulatory layer in late developmental stages. (4) ceRNA regulatory network (Figure 3A)—A miRNA “sponge” system centered around five long non-coding RNA (lncRNA) hubs [15], with node g1980.t1 (degree = 71) serving as the most central network hub. Functional perturbation of this node is predicted to induce network collapse and widespread dysregulation of target genes. These four regulatory layers collectively constitute a comprehensive post-transcriptional regulatory architecture spanning from coarse-grained (event-level) to fine-grained (single junction-level) and ultimately to system-level (ceRNA network-level) control. The observation of minimal transcriptional changes but highly active alternative splicing and junction usage during the stage D to E transition aligns with Nowrousian’s theory [22] of “chromatin remodeling and post-transcriptional regulation dominance in late developmental stages” across multiple fungal model species [22].

Random forest machine learning analysis (Figure 5B) revealed a metabolite signature with high statistical significance: among the top five most discriminative metabolites, 60% (including α-CEHC, 2,4-ditert-butylphenol, and carnosine) possess well-defined antioxidant biological functions. Carnosine, a β-alanyl-L-histidine dipeptide, exhibits dual physiological functions in pH buffering and free radical scavenging. Its prominent discriminative power (MDA = 1.72) strongly suggests that the coordinated regulation of acid-base homeostasis and redox balance represents a central regulatory mechanism during fungal development. Reactive oxygen species (ROS) serve a dual regulatory role in fungal development. At low concentrations, ROS act as crucial signaling molecules that promote cellular differentiation and morphogenesis, whereas at high concentrations, ROS trigger lipid peroxidation and protein oxidative damage, a phenomenon systematically documented across diverse fungal species [22]. The stage-specific accumulation pattern of antioxidant compounds during *B. xylophilus* development likely reflects a sophisticated dynamic ROS regulation strategy. During the rapid mycelial proliferation phase, excessive ROS are efficiently scavenged to protect dividing cells from oxidative damage; conversely, during fruiting body maturation, moderate oxidative stress is maintained to facilitate spore maturation while simultaneously inducing the activation of endogenous antioxidant defense systems, thereby ensuring that mature spores can withstand environmental oxidative challenges.

As an exceptionally rare non-mycorrhizal (saprophytic) species within the Boletaceae family, the metabolomic profile of *B. xylophilus* provides crucial biochemical foundations for its distinctive saprophytic lifestyle. Metabolic pathway analysis (Figure 5A) revealed significant enrichment of the ABC transporter system (KEGG map02010, involving 8 metabolites), reflecting a highly active transmembrane transport apparatus. The life history characteristics of saprophytic fungi necessitate the substantial secretion of extracellular degrading enzymes into the environment, coupled with the efficient absorption of degradation products, including monosaccharides, amino acids, and oligopeptides. This metabolic signature aligns remarkably with comparative genomic findings: the partial retention of lignocellulose-degrading enzyme gene families occurs in the genomes of *P. portentosus* and *B. xylophilus*, whereas these CAZyme genes have been nearly completely lost in ECM Boletaceae species [3]. Furthermore, significant enrichment of fatty acid biosynthesis pathways (KEGG map00061, 4 metabolites) and amino sugar and nucleotide sugar metabolism pathways (KEGG map00520, 4 metabolites) corresponds to the substantial demand for membrane biogenesis during rapid hyphal extension and the dynamic remodeling of cell wall architecture, respectively. Transcriptomic analysis through EggNOG functional annotation further revealed sustained high expression of multiple glycoside hydrolase family members, including GH61 (corresponding to fungal AA9 lytic polysaccharide monooxygenases, responsible for oxidative cleavage of crystalline cellulose), GH12, and GH16 families. Collectively, these enzymatic characteristics constitute the core metabolic strategy for complex carbon source acquisition in saprophytic fungi. The successful artificial cultivation of *B. xylophilus* demonstrates the translational application potential of its saprophytic metabolic capabilities in the edible mushroom industry. By integrating metabolomic and transcriptomic datasets, this study establishes a robust multi-omics foundation for subsequent molecular breeding initiatives, with specific applications including developmental cycle regulation, fruiting body yield enhancement, and nutritional quality optimization as key breeding objectives.

## 5. Conclusions

This study, based on an integrated systematic analysis of transcriptomic and untargeted metabolomic profiles, presents the first multidimensional molecular atlas of *B. xylophilus* throughout its developmental progression. Transcriptomic results show that the developmental program of this fungi exhibits nonlinear characteristics: the transition from mycelium (stage A) to primordium (stage C) represents the most intensive period of developmental reprogramming, during which 34.7% of all genes are differentially expressed, and the metabolomic model displays high sample discriminative power. In contrast, the transition from fruiting body maturation (stage D) to sporulation (stage E) constitutes a transcriptional fine-tuning phase, with only 3.4% of genes differentially expressed, although post-transcriptional regulation remains active. This pattern deviates from the conventional expectation that higher developmental stages correspond to greater molecular changes, suggesting a nonlinear progression of the developmental program. Competing endogenous RNA network analysis identified five key hub nodes of lncRNAs, with g1980.t1 exhibiting the highest degree value (71). Consensus clustering (k = 3) partitioned the samples into three developmental states: mycelium, primordium, and fruiting body.

At the metabolic level, pathway alterations were predominantly characterized by activation: the ratio of upregulated to downregulated metabolites in the stage between A and E comparison was 184:10, contrasting sharply with the relatively balanced transcriptomic ratio of upregulated to downregulated genes (988:928). This discrepancy suggests that the underlying mechanisms during *B. xylophilus* development may involve the global upregulation of translation efficiency, which in turn drives metabolic pathway activation, while downregulated genes, though low in abundance, may exert high regulatory potency. It should be noted with caution that the apparent predominance of upregulated over downregulated metabolites (18.4:1) may be partially attributable to the inherent detection bias of untargeted LC-MS/MS. Metabolites that increase in abundance generally exhibit higher ionization efficiency and are thus more readily detected and quantified, whereas those that decrease may fall below the detection limit and be missed. Therefore, this ratio should not be interpreted as a true biological stoichiometry, but rather as a semi-quantitative trend. Furthermore, the antioxidant defense system occupies a central role throughout development—60% of the top five discriminant metabolites, including α-CEHC, 2,4-di-tert-butylphenol, and carnosine, possess known antioxidant functions, indicating that maintenance of redox homeostasis is a metabolic hallmark across all developmental stages.

In the O2PLS analysis, the transcriptomic and metabolomic datasets mutually corroborated each other and converged on identical latent developmental signals. Pearson correlation analysis revealed that, among strongly positively correlated gene–metabolite pairs, functionally annotated genes and their corresponding metabolites formed significant positive correlation clusters, rendering these pairs as priority candidates for enzyme-to-product synthesis. The data points in the nine-quadrant plot were predominantly distributed along the diagonal from the upper right to the lower left, comprehensively depicting an anabolic chain of “transcriptional activation—increase in enzyme protein abundance—enhanced metabolic flux—accumulation of end products.” The number of gene–metabolite pairs in the lower-left quadrant was substantially smaller than that in the upper-right quadrant; this asymmetry implies that non-transcriptional regulatory layers make a non-negligible contribution to developmental metabolic regulation. WGCNA uncovered extensive gene–metabolite associations, suggesting that individual modules may participate in the developmental regulation of metabolites. The distribution of gene counts within modules reflected the hierarchical nature of developmental regulation, and the correspondence between modules and metabolite classes integrated scattered single-gene–metabolite statistical associations into a higher-level modular regulatory framework. Collectively, these results derived from transcriptome–metabolome integrative analyses constitute a candidate target system and regulatory network for subsequent functional validation, molecular breeding, and further studies on developmental regulation.

In summary, this study provides the first systemic multi-omics dataset for *B. xylophilus* in basidiomycete developmental biology; establishes a multi-layered molecular regulatory framework encompassing gene expression, metabolic phenotypes, and transcriptional regulation; and proposes several experimentally testable hypotheses, including the central role of antioxidant defense, regulatory functions of lncRNAs, metabolic pathway activation driven by the global upregulation of translation efficiency, and the multi-omics feature combination associated with a saprotrophic lifestyle. These findings not only deepen our multi-level understanding of the molecular mechanisms underlying fruiting body formation in macrofungi but also provide crucial omics references for the optimization of artificial cultivation and molecular breeding of this emerging edible and medicinal mushroom.

## Figures and Tables

**Table 1:** Statistics of DEGs across developmental stage comparisons.

Comparison	Total	Up	Down	Ratio	Genome %
A vs. B	1489	768	720	1.07	23.1
A vs. C	2233	1281	951	1.35	34.7
A vs. D	1835	1001	833	1.20	28.5
A vs. E	1917	988	928	1.06	29.8
B vs. C	1413	866	546	1.59	22.0
B vs. D	690	361	328	1.10	10.7
B vs. E	1117	561	555	1.01	17.4
C vs. D	956	391	564	0.69	14.9
C vs. E	1810	793	1016	0.78	28.1
D vs. E	217	59	158	0.37	3.4

**Table 2:** Statistics of differential splicing frequency at the junction level across developmental stage comparisons.

Comparison	Total SE Events	Significant SE Events	Total MXE Events	Significant MXE Events	Significant Rate of MXE (%)
A vs. B	664	7	9	4	44.4
A vs. C	908	3	13	7	53.8
A vs. D	724	7	12	8	66.7
A vs. E	698	7	13	8	61.5
B vs. C	648	2	7	4	57.1
B vs. D	429	0	7	3	42.9
B vs. E	412	2	6	4	66.7
C vs. D	705	3	10	2	20.0
C vs. E	688	5	10	4	40.0
D vs. E	470	0	9	3	33.3

**Table 3:** OPLS-DA supervised multivariate model results.

Stage Comparison	R^2^Y	Q^2^
A vs. B	0.972	0.899
A vs. C	0.997	0.940
A vs. D	0.823	0.758
A vs. E	0.981	0.947
B vs. C	0.989	0.564
B vs. D	0.870	0.747
B vs. E	0.987	0.903
C vs. D	0.863	0.744
C vs. E	0.984	0.909
D vs. E	0.872	0.582

## Data Availability

All summary data are provided in the results and Supplemental Files.

## Supplementary Materials

The following supporting information can be downloaded at https://www.mdpi.com/article/10.3390/biology15161343/s1. Figure S1: Principal component analysis (PCA) scatter plot of the transcriptome across five developmental stages (A to E) of *B. xylophilus*; Figure S2: Bubble plot of EggNOG KO enrichment analysis for differentially expressed genes across five developmental stages (A to E) of *B. xylophilus* (A vs. B, B vs. C, C vs. D, D vs. E); Figure S3: Heatmap of the confusion matrix for random forest (RF) model classification across five developmental stages (A to E) of *B. xylophilus*; Figure S4: Scale-free topology fit index (R^2^) and mean connectivity corresponding to different soft-thresholding powers in WGCNA network construction for *B. xylophilus*; Figure S5: PCA score scatter plot of the metabolome across five developmental stages (A to E) of *B. xylophilus*; Figure S6: Volcano plots of differential metabolites in the metabolome across five developmental stages (A to E) of *B. xylophilus* (A vs. B, B vs. C, C vs. D, D vs. E); Figure S7: Z score normalized expression heatmap of the top 200 differential metabolites across five developmental stages (A to E) of *B. xylophilus*; Figure S8: Variance decomposition of each component in the O2PLS model for *B. xylophilus*; Figure S9: Scale free topology fit index (R^2^) and mean connectivity corresponding to different soft thresholding powers in WGCNA network construction for *B. xylophilus*; Figure S10: Dendrogram of WGCNA gene clustering and co-expression module color assignments in *B. xylophilus*. Table S1: Comparative performance of various machine learning models evaluated under 5-fold cross-validation, training set, and test set for the five developmental stages (A–E) of *B. xylophilus*; Table S2: Assignment of module colors to individual genes identified in the WGCNA co-expression network; Table S3: List of hub genes for each co-expression module in the WGCNA network of *B. xylophilus*; Table S4. List of lncRNA-mRNA interaction pairs identified in the ceRNA regulatory network associated with developmental stages of *B. xylophilus*; Table S5: Differential metabolites in *B. xylophilus* across developmental stages; Table S6: List of all metabolic features detected by untargeted metabolomics and their RF importance scores (MDA) in *B. xylophilus*.
