# Supplementary material for: Integrated Multi-Omics Reveals the Developmental Programs and Metabolic Regulation of the Rare Edible Fungus Buchwaldoboletus xylophilus
Source: Biology (Basel). 2026 Aug 8;15(16):1343. doi: 10.3390/biology15161343 (PMC13509997; doi:10.3390/biology15161343)
Supplement: Supplementary file 1 [file biology-15-01343-s001.zip › Supplemental Material.pdf]

Integrated Multi-Omics Reveals the Developmental Programs and Metabolic Regulation of the Rare Edible Fungus *Buchwaldoboletus xylophilus*

Yuying Liu [1,2], Zhiyuan Jia [1], Teng Jiang [3], Na Zhang [1,2], Lixiao Song [1,2], Jin Zhang [1,2], Xiaolei Wan [1], Haiqiang Wang [1], Can Du [4], Daoyin Shen [3], Minglei Li [1,2,*] and Jianzhao Qi [1,2,3,*]

[1] Shaanxi Key Laboratory of Natural Products & Chemical Biology, College of Chemistry & Pharmacy, Northwest A&F University, Yangling, Xianyang 712100, China
[2] Center of Edible Fungi, Northwest A&F University, Yangling, Xianyang 712100, China
[3] Yangling Zhijun Fungi Biotechnology Engineering Co., Ltd., Yangling 712100, China
[4] Shaanxi Agriculture and Forestry Technology University, Yangling, Xianyang 712100, China
* Correspondence: mlli@nwafu.edu.cn (M.L.); qjz@nwafu.edu.cn (J.Q.)

Content

Figure S1. PCA scatter plot of the transcriptome across five developmental stages (A–E) of *B. xylophilus.* 1
Figure S2. Bubble plot of EggNOG KO enrichment analysis for DEGs across five developmental stages (A to E) of *B. xylophilus* (A vs B, B vs C, C vs D, D vs E)........................ 3
Figure S3. Heatmap of the confusion matrix for random forest (RF) model classification across five developmental stages (A–E) of *B. xylophilus*. 4
Figure S4. Scale-free topology fit index (R^2) and mean connectivity corresponding to different soft-thresholding powers in WGCNA network construction for *B. xylophilus*. 5
Figure S5. PCA score scatter plot of the metabolome across five developmental stages (A to E) of *B. xylophilus*.. 6
Figure S6. Volcano plots of differential metabolites in the metabolome across five developmental stages (A to E) of *B. xylophilus* (A vs B, B vs C, C vs D, D vs E)........................ 8
Figure S7. Z-score-normalized expression heatmap of the top 200 differential metabolites across five developmental stages (A to E) of *B. xylophilus*.. 9
Figure S8. Variance decomposition of each component in the O2PLS model for *B. xylophilus*. 10
Figure S9. Scale-free topology fit index (R^2) and mean connectivity corresponding to different soft-thresholding powers in WGCNA network construction for *B. xylophilus*. ..11
Figure S10. Figure S10. Dendrogram of WGCNA gene clustering and co-expression module color assignments in *B. xylophilus*.. 12

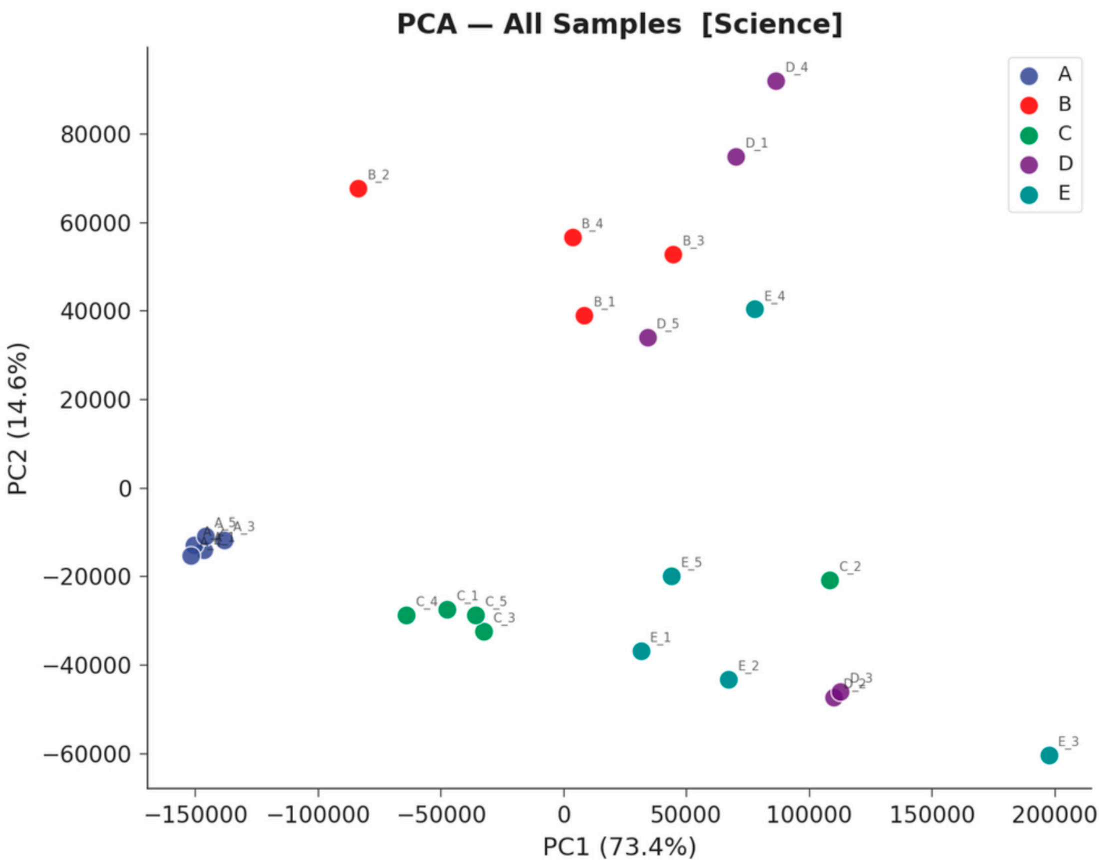

Figure S1. PCA scatter plot of the transcriptome across five developmental stages (A–E) of *B. xylophilus.*

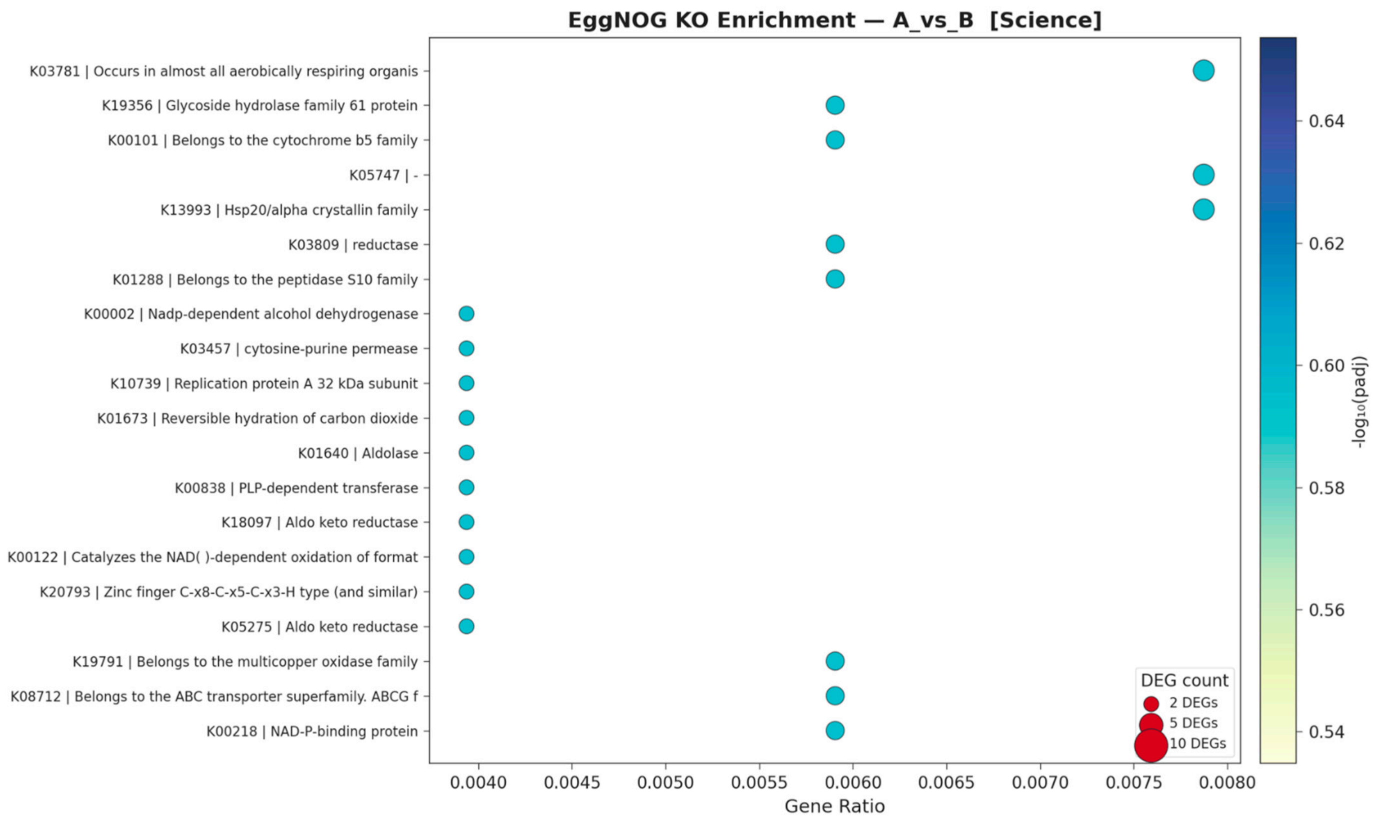
EggNOG KO Enrichment — A_vs_B [Science]
K03781 | Occurs in almost all aerobically respiring organis
K19356 | Glycoside hydrolase family 61 protein
K00101 | Belongs to the cytochrome b5 family
K05747 | -
K13993 | Hsp20/alpha crystallin family
K03809 | reductase
K01288 | Belongs to the peptidase S10 family
K00002 | Nadp-dependent alcohol dehydrogenase
K03457 | cytosine-purine permease
K10739 | Replication protein A 32 kDa subunit
K01673 | Reversible hydration of carbon dioxide
K01640 | Aldolase
K00838 | PLP-dependent transferase
K18097 | Aldo keto reductase
K00122 | Catalyzes the NAD( )-dependent oxidation of format
K20793 | Zinc finger C-x8-C-x5-C-x3-H type (and similar)
K05275 | Aldo keto reductase
K19791 | Belongs to the multicopper oxidase family
K08712 | Belongs to the ABC transporter superfamily. ABCG f
K00218 | NAD-P-binding protein
DEG count
2 DEGs
5 DEGs
10 DEGs
0.0040
0.0045
0.0050
0.0055
0.0060
0.0065
0.0070
0.0075
0.0080
Gene Ratio
0.64
0.62
0.60
0.58
0.56
0.54
-log₁₀(padj)

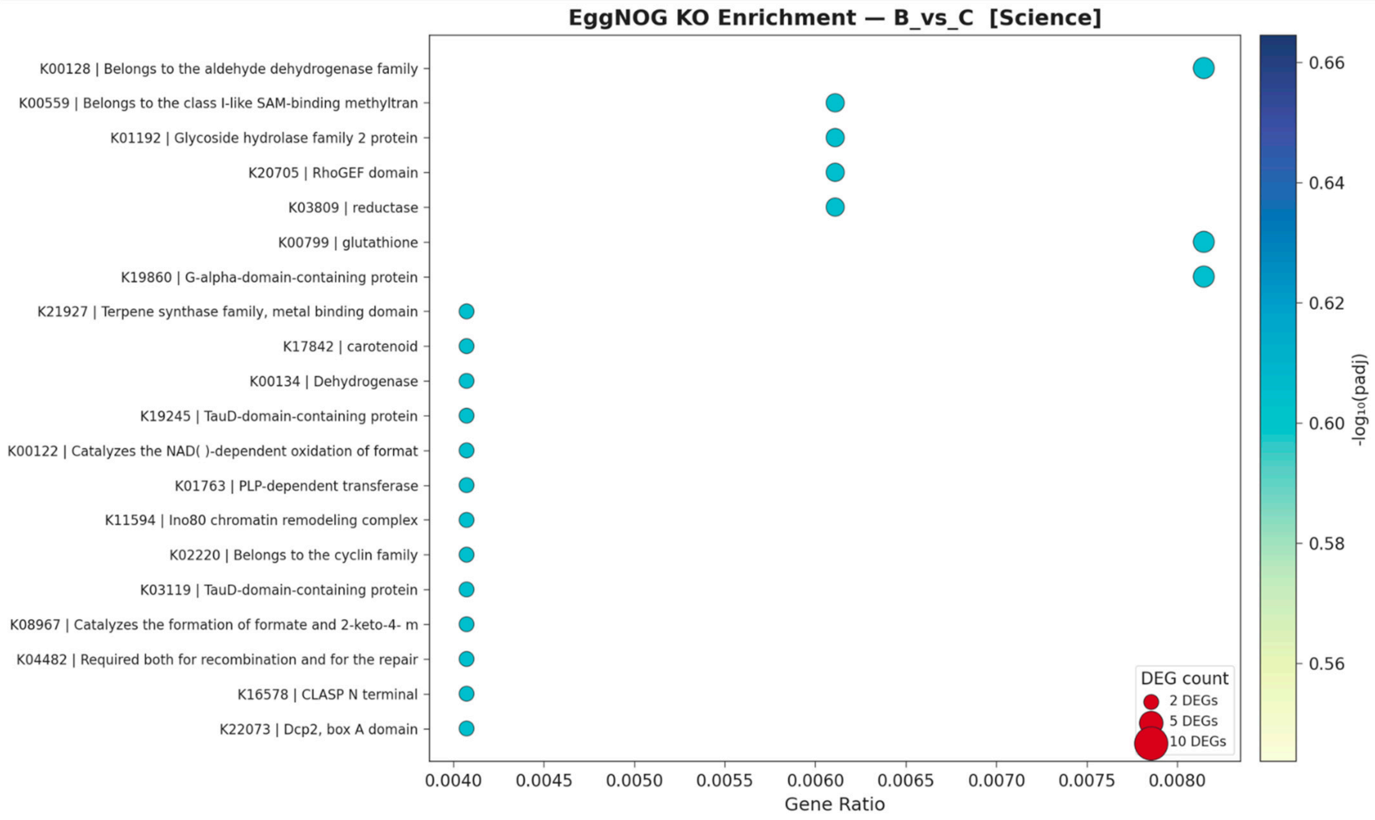
EggNOG KO Enrichment — B_vs_C [Science]
K00128 | Belongs to the aldehyde dehydrogenase family
K00559 | Belongs to the class I-like SAM-binding methyltran
K01192 | Glycoside hydrolase family 2 protein
K20705 | RhoGEF domain
K03809 | reductase
K00799 | glutathione
K19860 | G-alpha-domain-containing protein
K21927 | Terpene synthase family, metal binding domain
K17842 | carotenoid
K00134 | Dehydrogenase
K19245 | TauD-domain-containing protein
K00122 | Catalyzes the NAD( )-dependent oxidation of format
K01763 | PLP-dependent transferase
K11594 | Ino80 chromatin remodeling complex
K02220 | Belongs to the cyclin family
K03119 | TauD-domain-containing protein
K08967 | Catalyzes the formation of formate and 2-keto-4- m
K04482 | Required both for recombination and for the repair
K16578 | CLASP N terminal
K22073 | Dcp2, box A domain
DEG count
2 DEGs
5 DEGs
10 DEGs
0.0040
0.0045
0.0050
0.0055
0.0060
0.0065
0.0070
0.0075
0.0080
Gene Ratio
0.66
0.64
0.62
0.60
0.58
0.56
-log₁₀(padj)

E).

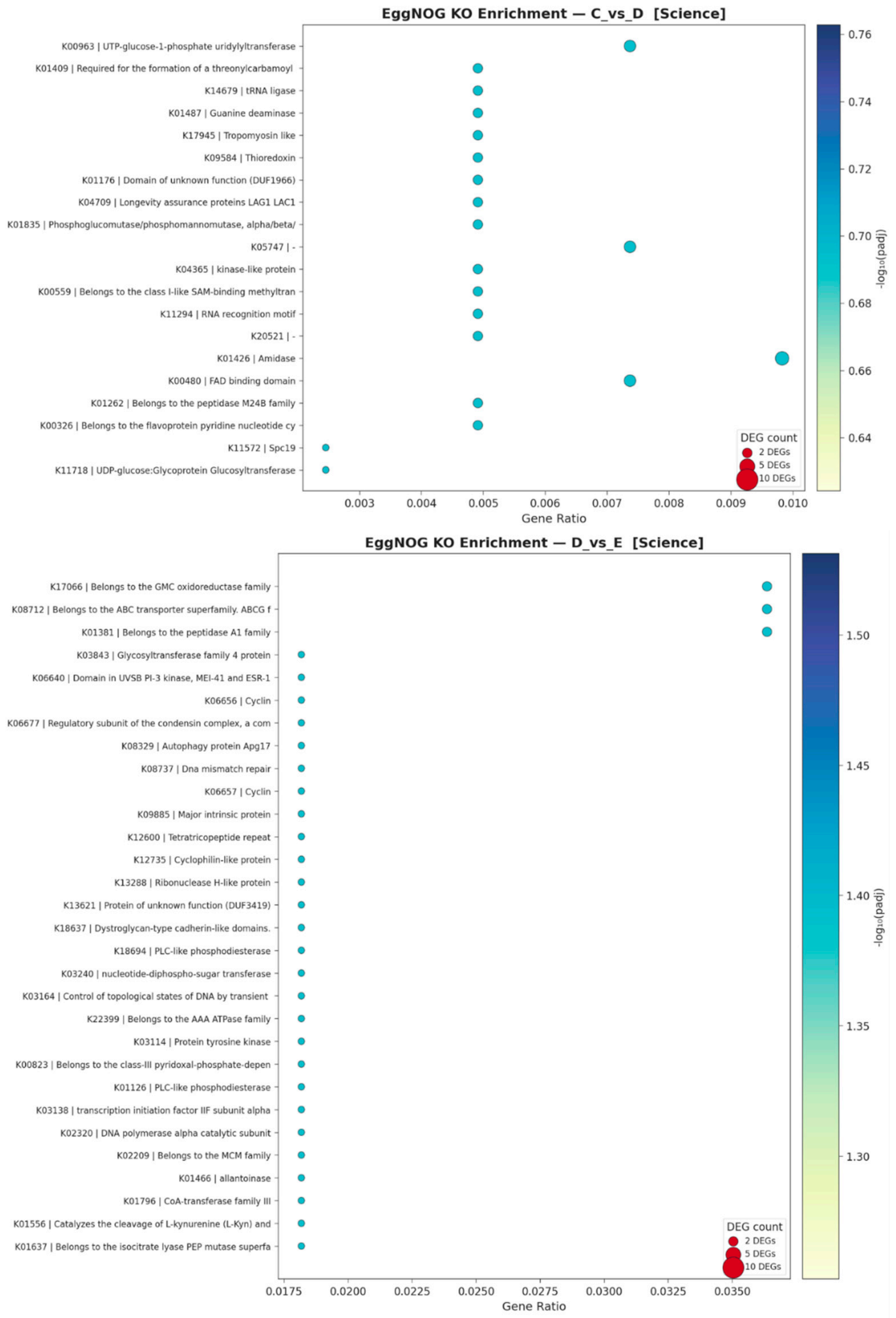

Figure S2. Bubble plot of EggNOG KO enrichment analysis for DEGs across five developmental stages (A to E) of *B. xylophilus* (A vs B, B vs C, C vs D, D vs E).

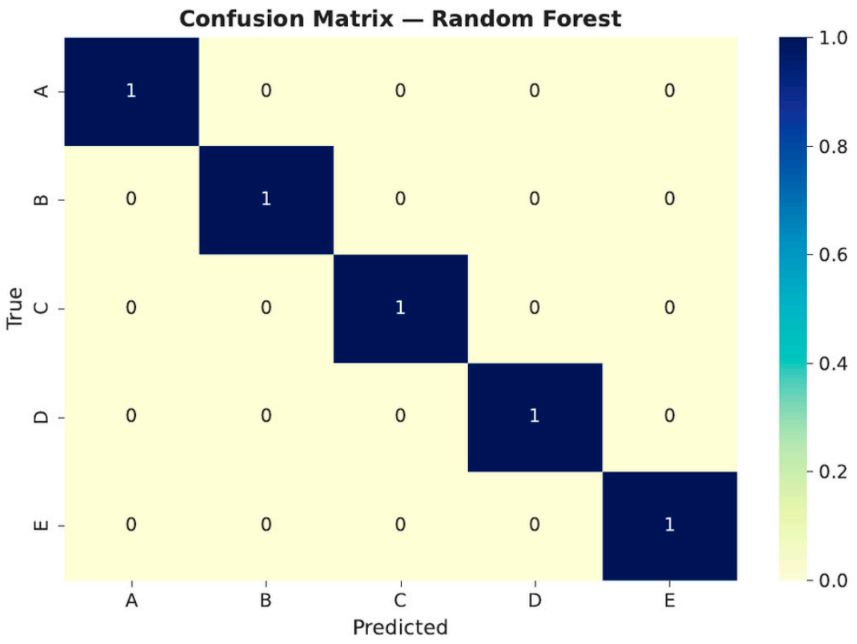

Figure S3. Heatmap of the confusion matrix for random forest (RF) model classification across five developmental stages (A–E) of *B. xylophilus*.

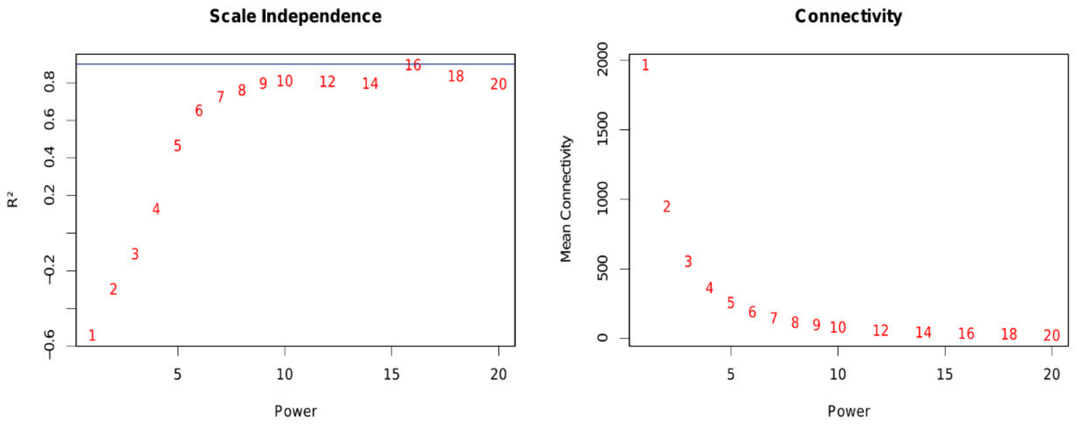

Figure S4. Scale-free topology fit index (R^2) and mean connectivity corresponding to different soft-thresholding powers in WGCNA network construction for *B. xylophilus*.

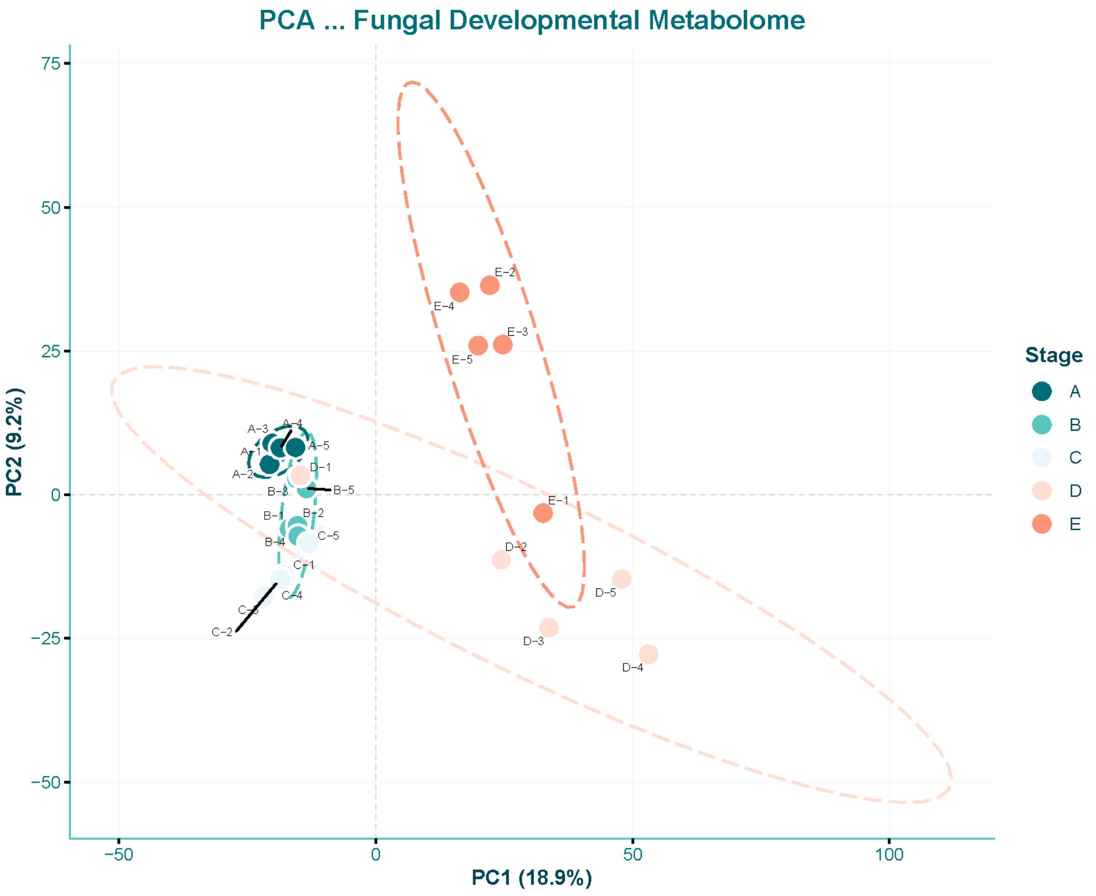

Figure S5. PCA score scatter plot of the metabolome across five developmental stages (A to E) of *B. xylophilus*.

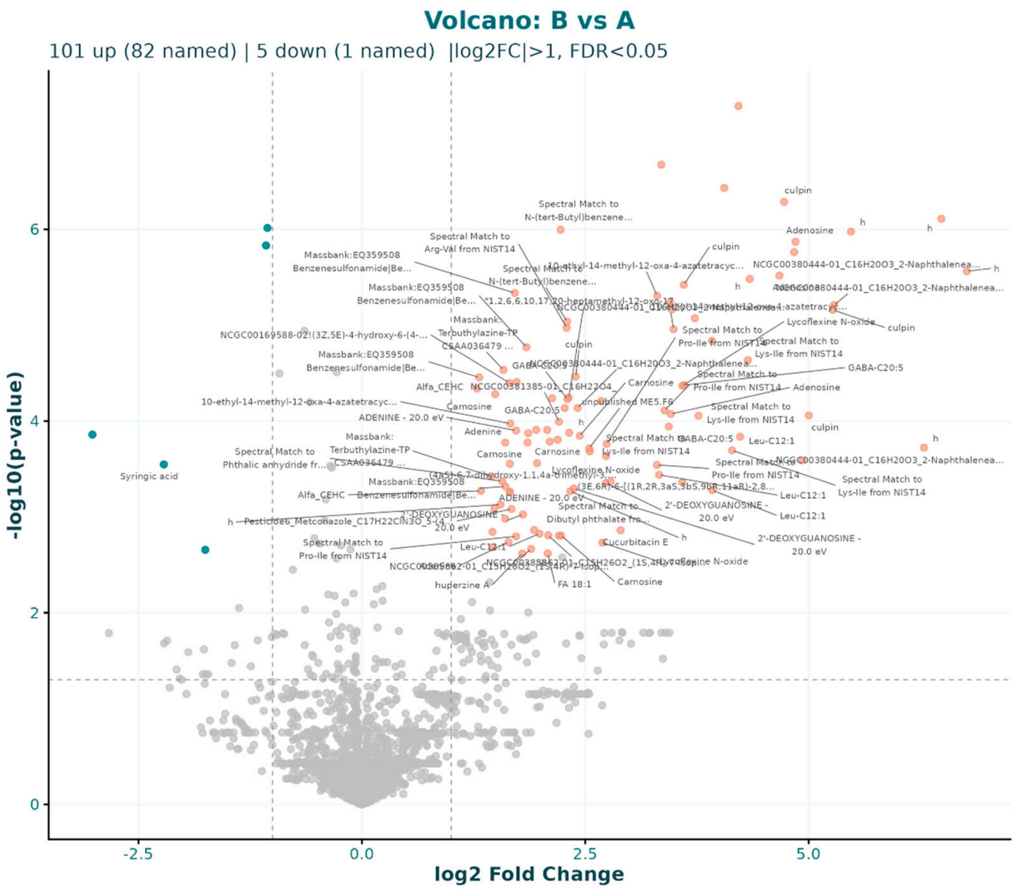
Volcano: B vs A
101 up (82 named) | 5 down (1 named) |log2FC|>1, FDR<0.05
-log10(p-value)
log2 Fold Change
-2.5
0.0
2.5
5.0
0
2
4
6
Syringic acid

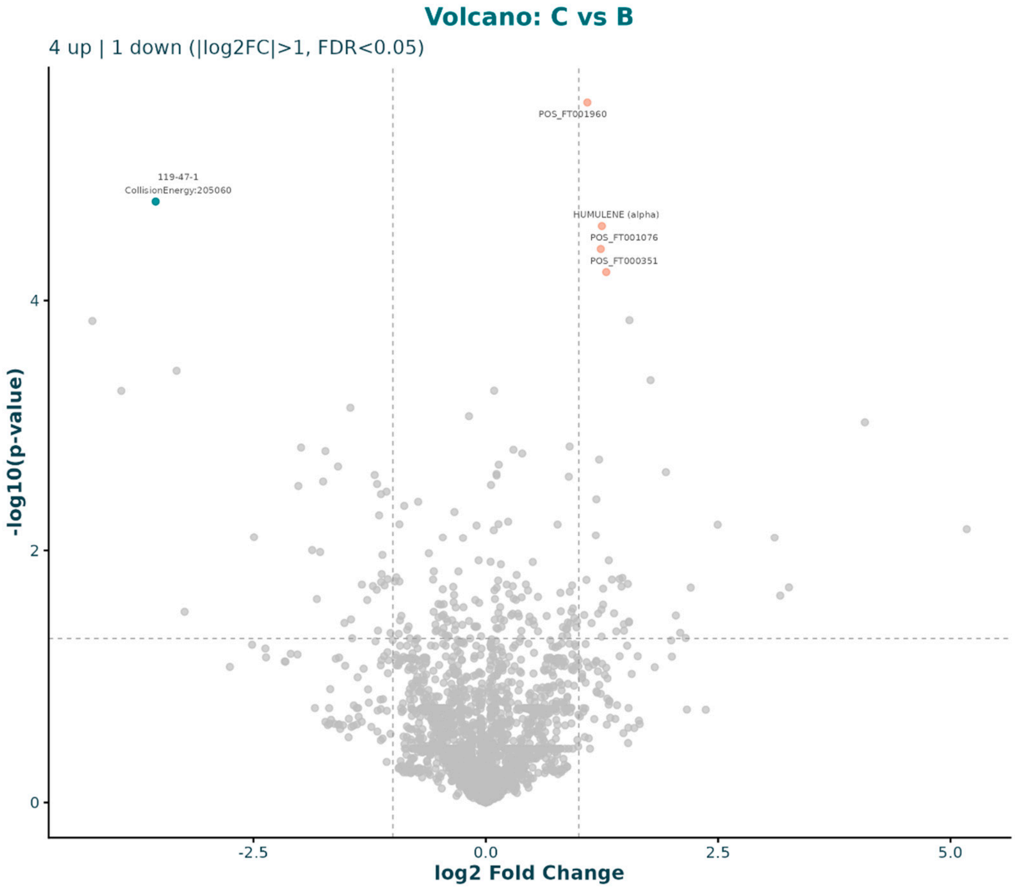
Volcano: C vs B
4 up | 1 down (|log2FC|>1, FDR<0.05)
-log10(p-value)
log2 Fold Change
-2.5
0.0
2.5
5.0
0
2
4
POS_FT001960
119-47-1
CollisionEnergy:205060
HUMULENE (alpha)
POS_FT001076
POS_FT000351

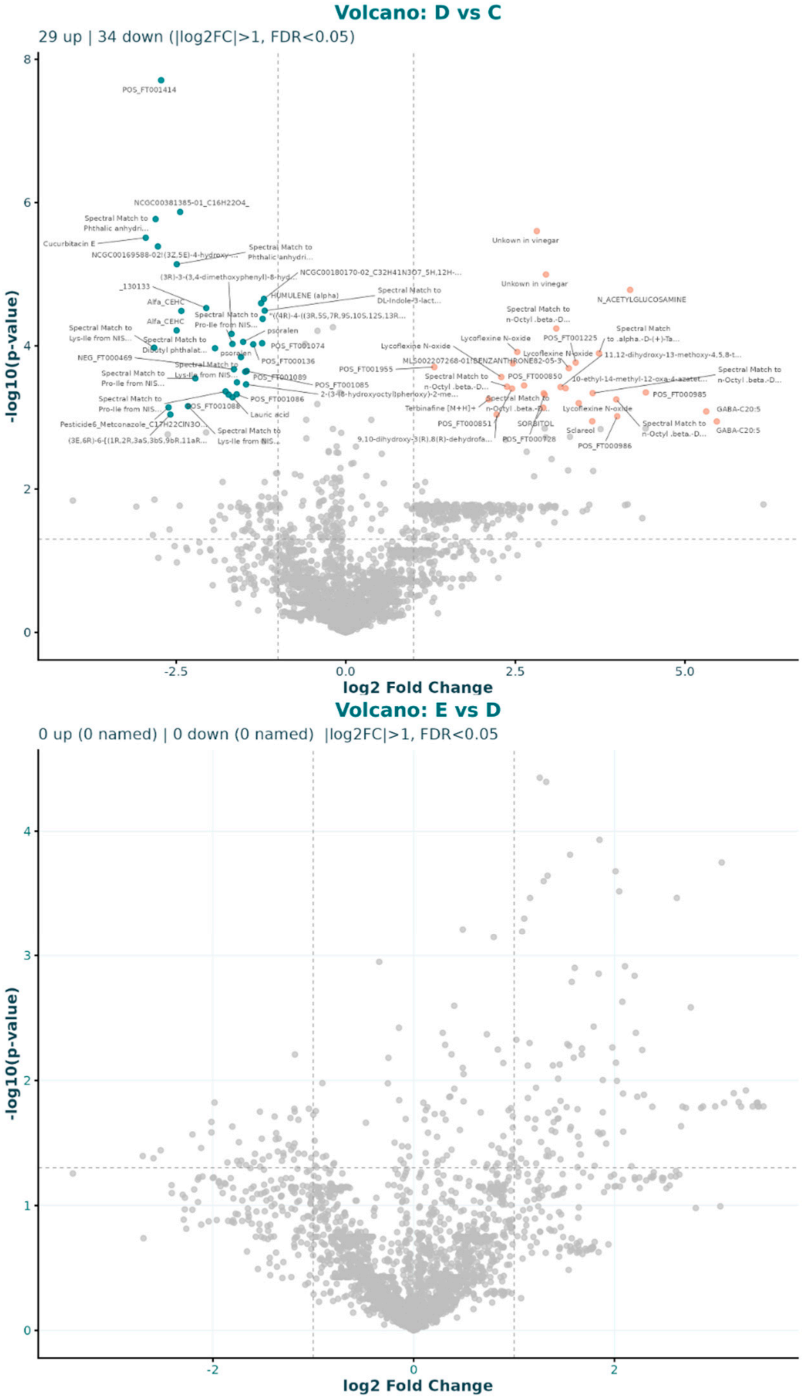

Figure S6. Volcano plots of differential metabolites in the metabolome across five developmental stages (A to E) of *B. xylophilus* (A vs B, B vs C, C vs D, D vs E).

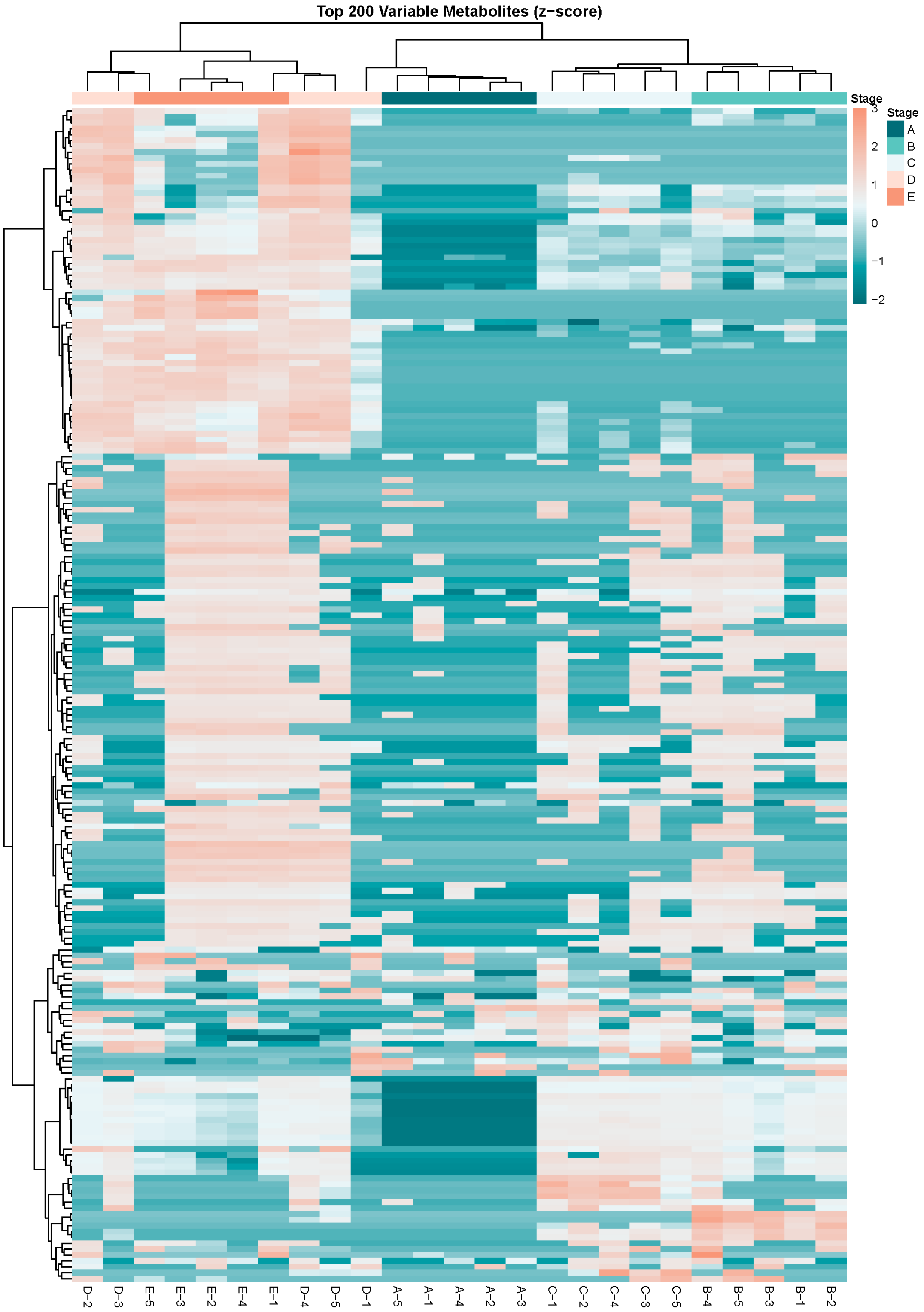

Figure S7. Z-score-normalized expression heatmap of the top 200 differential metabolites across five developmental stages (A to E) of *B. xylophilus*.

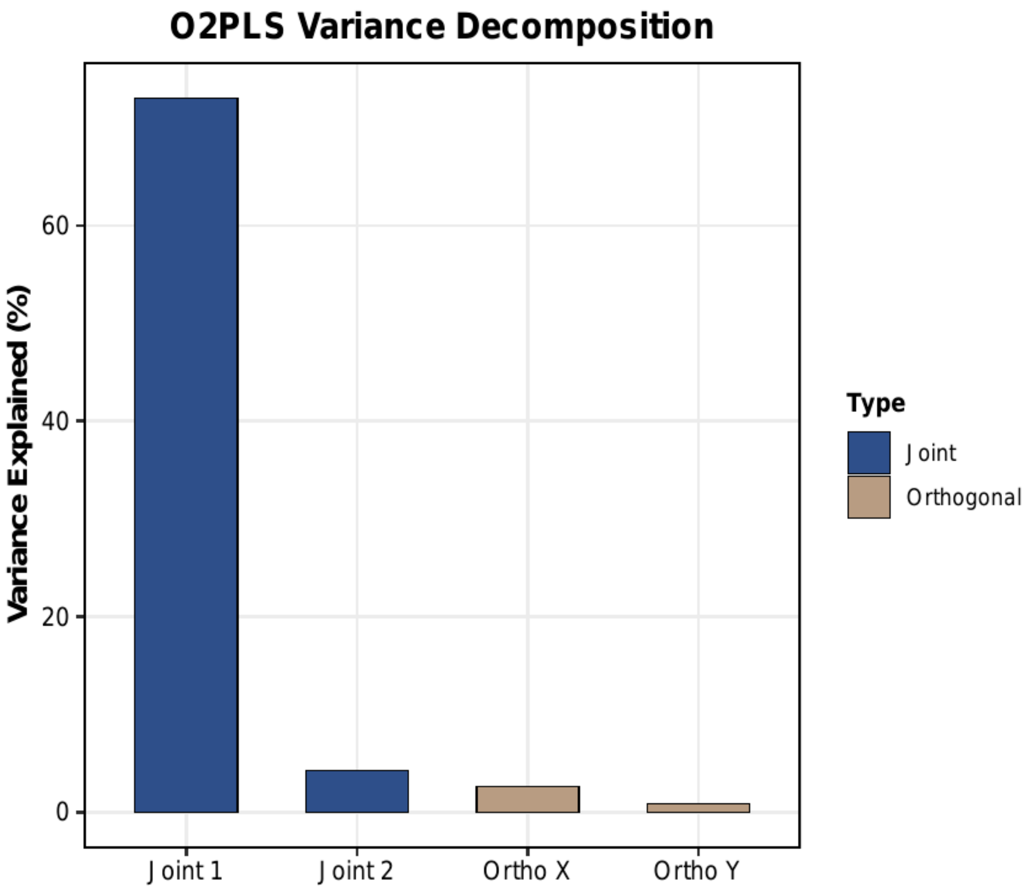

Figure S8. Variance decomposition of each component in the O2PLS model for *B. xylophilus*.

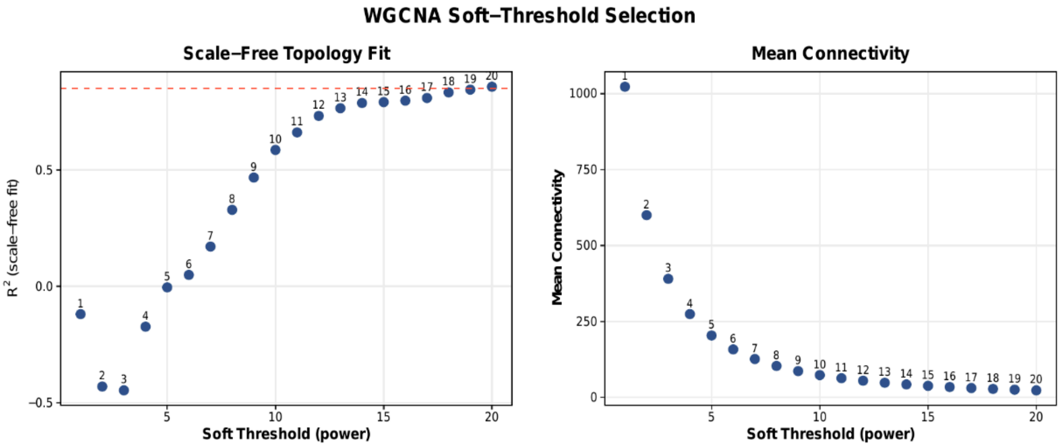

Figure S9. Scale-free topology fit index (R^2) and mean connectivity corresponding to different soft-thresholding powers in WGCNA network construction for *B. xylophilus*.

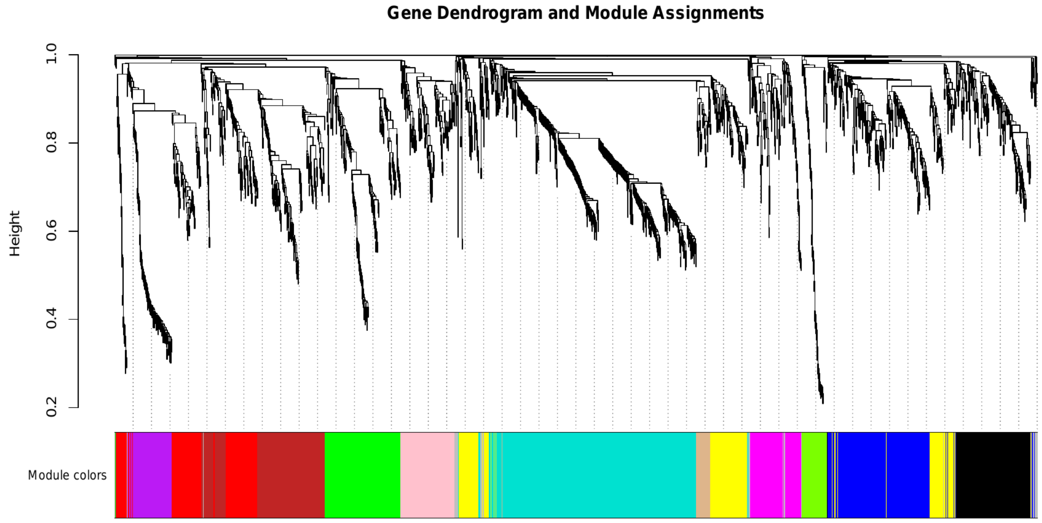

Figure S10. Dendrogram of WGCNA gene clustering and co-expression module color assignments in *B. xylophilus*.
